# Anchoring PdO_x_ clusters on defective alumina for improved catalytic methane oxidation

**DOI:** 10.1038/s41467-024-50216-0

**Published:** 2024-08-01

**Authors:** Xiang Yu, Nina S. Genz, Rafael G. Mendes, Xinwei Ye, Florian Meirer, Maarten Nachtegaal, Matteo Monai, Bert M. Weckhuysen

**Affiliations:** 1 Inorganic Chemistry and Catalysis, Debye Institute for Nanomaterials Science & Institute for Sustainable and Circular Chemistry, Utrecht University, Universiteitsweg 99, CG Utrecht, 3584 The Netherlands; 2 Paul Scherrer Institute, Forschungsstrasse 111, Villigen, 5232 Switzerland; 3 Soft Condensed Matter, Debye Institute for Nanomaterials Science, Utrecht University, Princetonplein 5, CC Utrecht, 3584 The Netherlands; 4 Interfaces, Confinement, Matériaux et Nanostructures, CNRS-Orléans, 1b rue de la Férollerie, Orléans, 45071 France

**Keywords:** Heterogeneous catalysis, Catalytic mechanisms, Catalyst synthesis

## Abstract

Evolution of the Pd active centers in size and spatial distribution leads to an irreversible deactivation in many high-temperature catalytic processes. This research demonstrates the use of a defective alumina (Al_2_O_3-x_) as catalyst support to anchor Pd atoms and suppress the growth of Pd clusters in catalytic methane oxidation. A combination of operando spectroscopy and density functional theory (DFT) calculations provide insights into the evolution of Pd species and reveals distinct catalytic methane oxidation mechanisms on Pd single atoms, clusters, and nanoparticles (NPs). Among these Pd species, the cluster active centers are found to be the most favorable participants in methane oxidation due to their high dispersion, high content of Pd^2+^ oxidation state, and resistance to deactivation by carbonates, bicarbonates, and water. The Pd/Al_2_O_3-x_ catalyst shows increased stability with respect to a Pd/Al_2_O_3_ counterpart during simulated aging in alternating reducing and oxidizing conditions due to stronger interactions with the support. This study demonstrates that defect engineering of non-reducible supports can constrain the evolution of active centers, which holds promising potential for widespread utilization across diverse industrial catalytic processes, including various hydrogenation and oxidation reactions.

## Abstract (web summary)

The evolution of Pd active centers in size and spatial distribution often leads to irreversible deactivation in many high-temperature catalytic processes. Here the authors demonstrate the use of defective alumina (Al_2_O_3-x_) as a catalyst support to anchor Pd atoms and suppress the growth of Pd clusters in catalytic methane oxidation.

## Introduction

Natural gas has been extensively used in power generation and other heating applications because of its abundant global storage, high knock resistance, and low carbon/hydrogen ratio^1–3^. However, methane (CH_4_) has a climate forcing effect 87 times bigger than carbon dioxide (CO_2_) over a 25-year period^4^, and thus high specific activity is needed at low temperatures to avoid unwanted CH_4_ emission, especially during engine start-up^5,6^. Hence, CH_4_ catalytic oxidation is especially important in controlling harmful green-house gas emissions in exhaust after treatments of natural gas vehicles, which are emerging alternatives to gasoline^6–8^. Among the explored catalyst materials, Pd/Al_2_O_3_  is considered as the most desirable commercial catalyst for CH_4_ oxidation under lean-burn conditions because of its high activity and relatively low cost^6,8^. However, the gradual deactivation of Pd/Al_2_O_3_ catalysts over long periods of use remains a major problem for their practical implementation^3,8–10^.

Previous studies have revealed many reasons for the deactivation of Pd/Al_2_O_3_ catalyst materials^3^. For instance, the active centers may be poisoned by SO_x_, H_2_O and carbonaceous species^11–14^. Such deactivation is non-permanent. Removal of surface poisons by adding periodic activation steps allow the catalyst to be regenerated or rejuvenated^15,16^. Permanent deactivation is typically due to the growth of Pd clusters into bigger Pd particles at high temperatures and during redox cycling, for which a practical solution has been rarely reported^7,10^. A practical way to suppress the nanoparticle growth is to modify the interaction between support and metal species^17,18^. On the one hand, strong interactions secure the high dispersion degree of the active center that allows more metal atoms to participate in a reaction, while on the other hand the weak interactions lead to sintering and aggregation of metal NPs.

Although the conventional view suggests that only weak interactions exist between precious metals and alumina, a growing amount of experimental evidence has indicated that traces of so-called pentacoordinate aluminum (Al^V^) appearing over γ-Al_2_O_3_ during thermal treatment can anchor precious metal ions^19^. However, due to the limited concentration of Al^v^ sites in γ-Al_2_O_3_, this interaction is mostly neglected in catalysis research. Of course, we should realize the distorted tetrahedral Al^IV^ sites, which are typically found in highly ordered alumina and silica-alumina, may also interfere with the identification of Al^V^ ^20^.

In this work, we have developed a synthetic route to increase the concentration of Al^V^ in amorphous alumina (Al_2_O_3-x_), having a high specific surface area and micro-mesoporous pore structure. Such support was used to anchor surface Pd species, preventing their aggregation and resulting in a more active and stable catalyst for CH_4_ oxidation. High-angle annular dark-field scanning transmission electron microscopy (HAADF-STEM) and operando quick-extended X-ray absorption fine structure (QEXAFS) were used to study the evolution of Pd species on defective Al_2_O_3-x_ and γ-Al_2_O_3_ supports during CH_4_ oxidation. In order to reveal the relation between deactivation and the evolution of Pd species, the intrinsic activities and CH_4_ catalytic oxidation mechanism over Pd nanoparticles (NPs), clusters and single-atom species present on Pd/Al_2_O_3_ and Pd/Al_2_O_3-x_ were studied with the combination of operando Fourier transform infrared (FT-IR) spectroscopy and density functional theory (DFT) calculations.

## Results and discussion

### Evolution of Pd species over γ-Al_2_O_3_ and defective Al_2_O_3-x_ during CH_4_ oxidation

**Fig. 1:**
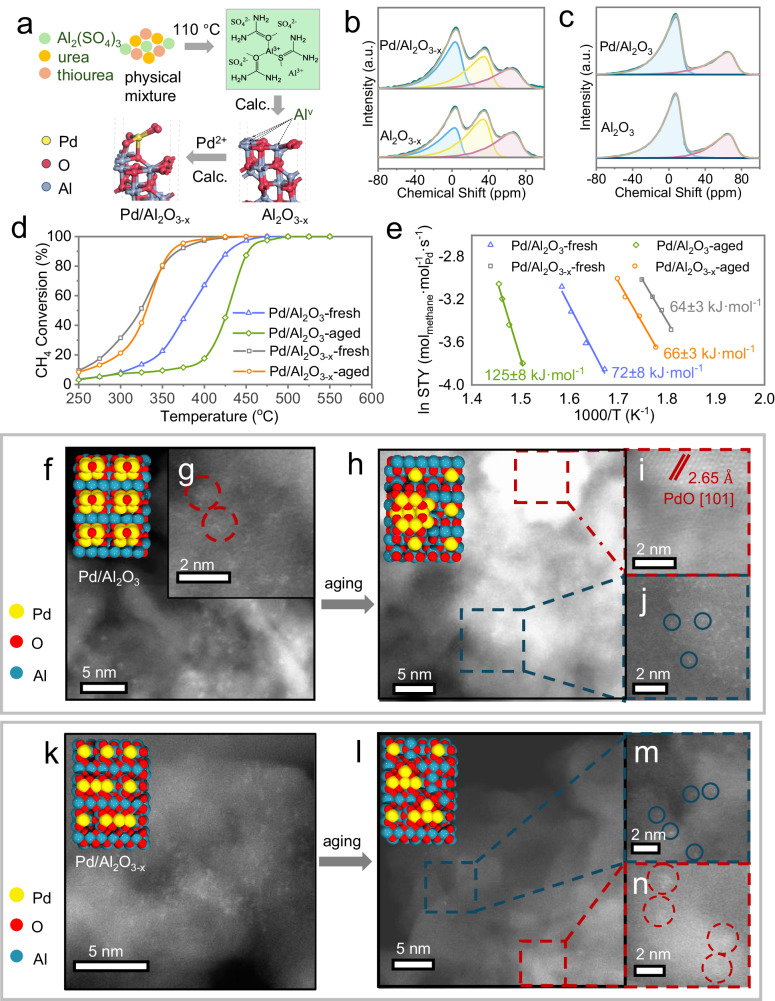
Stabilizing active palladium species on defective alumina sites. **a** Schematic illustration of the synthesis of defective alumina (Al_2_O_3-x_) and Pd/Al_2_O_3-x_. ^27^Al solid state nuclear magnetic resonance (ssNMR) spectra and respective fitting for Al_2_O_3-x_ (**b** bottom), Pd/Al_2_O_3-x_ (**b** top), γ-Al_2_O_3_ (**c** bottom) and Pd/Al_2_O_3_ (**c** top) The deconvoluted peaks, denoted by blue, yellow, and red colors, respectively represent the tetrahedral, pentahedral, and octahedral coordination Al species. **d** Light-off curves for catalytic methane  (CH_4_) oxidation over fresh and aged Pd/Al_2_O_3_ and Pd/Al_2_O_3-x_ catalysts under reaction conditions (500 °C, 1.5 h). Reaction conditions: CH_4_:O_2_:He = 2:8:90, 50 mg catalyst, GHSV = 60,000 h^−1^. **e** Arrhenius plots of the fresh and aged Pd catalysts. The high-angle annular dark-field scanning transmission electron microscopy (HAADF-STEM) images of Pd/Al_2_O_3_-fresh (**f**, **g**); Pd/Al_2_O_3_-aged (**h**–**j**); Pd/Al_2_O_3-x_-fresh (**k**) and Pd/Al_2_O_3-x_-aged (**l**–**n**). The red and blue dashed boxes represent respectively the regions where PdO_x_ clusters/nanoparticles (NPs) and single atoms are present on the catalysts. Pd clusters are marked with the red rings, while Pd single atoms are marked with blue rings.

The synthesis of defective alumina was realized by pyrolysis of aluminum sulfate dissolved in a deep eutectic solvent composed of urea and thiourea (Fig. 1a, see Supplementary information, Section 1 for details). Pd/Al_2_O_3_ and Pd/Al_2_O_3-x_ catalysts with a nominal Pd loading of 1 wt%, were prepared by the incipient wetness impregnation (IWI) method followed by calcination at 400 °C for 2 h (see Supplementary information, Sections 1.3 and 4 for details). The ^27^Al solid-state nuclear magnetic resonance (ssNMR) results revealed the presence of three prominent peaks at around 5, 38 and 70 ppm over Al_2_O_3-x_ (Fig. 1b), which can be ascribed to Al^3+^ species in tetrahedral (AlO_4_ at 2–8 ppm), pentahedral (AlO_5_ at 36–40 ppm) and octahedral (AlO_6_ at 68-70 ppm) coordination, respectively^19,21^. To quantify the different Al species, the ^27^Al ssNMR spectra were fitted by three components using a simple Czjzek model^22,23^. After loading of Pd, a decrease in the mole percent content of both Al^V^ (from 40 to 34%) and Al^IV^ (from 26 to 24%) on the Al_2_O_3-x_ support was observed, accompanied by an elevation in the content of Al^VI^. While previous research has evidenced the role of Al^V^ in the anchoring of isolated noble metals^19^, the observed decrease in the relative content of Al^IV^ species here may also indicate binding of Pd atoms on Al^IV^. One possible explanation is the occurrence of localized surface reconstruction caused by the interaction between distorted Al^IV^ species and Pd through oxygen bridging (Fig. 1a), leading to the conversion of tetrahedrally coordinated Al to an octahedral coordination structure. Furthermore, the total quantity of consumed Al^V^ and Al^IV^ sites was eight times greater than the amount of loaded Pd, which may be attributed to: (a) the fixation of Pd atoms requiring the consumption of multiple Al^V^ and Al^IV^ species^24^ (Fig. 1a and Supplementary Information, Section 8.1 for details); (b) the introduction of Pd causing surface restructuring of amorphous alumina due to strong metal-support interaction^19,21^; and (c) the presence of trace amounts of water in the impregnation solution resulting in hydroxylation of Al^V^ and distorted Al^IV^ sites. Notably, the remaining Al^V^ was still sufficient to immobilize an equal or even greater amount of Pd species or to restrict the mobility of Pd during the reaction^25^. In contrast, ^27^Al NMR spectroscopy did not detect Al^V^ sites on γ-Al_2_O_3_ at room temperature, regardless of the presence of Pd species (Fig. 1c). The proportion between Al^IV^ and Al^VI^ also remained invariant on γ-Al_2_O_3_ before and after the loading process.

The catalytic activity of Pd/Al_2_O_3_ and Pd/Al_2_O_3-x_ for the complete oxidation of CH_4_ was evaluated using a fixed-bed downflow reactor. The light-off curves (Fig. 1d) for CH_4_ conversion show that the fresh Pd/Al_2_O_3-x_ exhibited higher catalytic activity (reaching 90% methane conversion at T_90_ = 367 °C) compared to Pd/Al_2_O_3_ (T_90_ = 429 °C). After ageing for 1.5 h at 500 °C with GHSV of 120,000 h^−1^, Pd/Al_2_O_3-x_-aged lost some of its low-temperature activity, but the catalytic activity was improved at relatively high temperatures (Fig. 1d). In contrast, Pd/Al_2_O_3_-aged showed noticeably lower activity compared to Pd/Al_2_O_3_-fresh across all temperatures. The corresponding Arrhenius plots (Fig. 1e) demonstrated that Pd/Al_2_O_3_-aged possesses a higher apparent activation energy (125 kJ/mol) in comparison to Pd/Al_2_O_3_-fresh (72 kJ/mol), Pd/Al_2_O_3-x_-fresh (64 kJ/mol) and Pd/Al_2_O_3-x_-aged (66 kJ/mol). Based on the apparent activation energy values, it can be inferred that the active centers over Pd/Al_2_O_3-x_ exhibit higher intrinsic activity than those on Pd/Al_2_O_3_, and that they undergo negligible changes in the aging process. On the other hand, the ageing process significantly changed the active centers of Pd/Al_2_O_3_, generating intrinsically less active Pd species. The intercepts of the fitted Arrhenius plots, in first approximation related to the quantity of active sites, provide additional evidence that fresh Pd/Al_2_O_3-x_ possesses a higher quantity of accessible active centers than Pd/Al_2_O_3_, whereas ageing causes the loss of active species on both catalysts^26^. Furthermore, during stability testing at 500 °C, Pd/Al_2_O_3_ gradually deactivated over time, consistent with previous studies^10^, while Pd/Al_2_O_3-x_ remained relatively active for 15 h (Supplementary Information, Section 5 for details). Altogether, the results obtained suggest that Pd species on Al_2_O_3-x_ were stabilized against thermally-induced deactivation processes during CH_4_ oxidation.

We performed HAADF-STEM to obtain atomically resolved images of Pd species present on Pd/Al_2_O_3_ and Pd/Al_2_O_3-x_, for both fresh and aged catalysts. As shown in Fig. 1f, g, fresh Pd/Al_2_O_3_ consists mainly of small Pd NPs of 1–2 nm. After 1.5 h of ageing under reaction conditions, a fraction of the Pd species transformed into isolated Pd ions (Fig. 1j), potentially anchored on the generated Al^V^ sites during reaction^19^. Meanwhile the remaining Pd species sintered and agglomerated into large PdO NPs (Fig. 1h, i). In contrast, the Pd on the fresh Pd/Al_2_O_3-x_ catalyst consisted mainly of Pd single-atom and low-nuclear PdO_x_ clusters (Fig. 1k). After ageing (Fig. 1l), a portion of the Pd species maintained their single-atom feature (Fig. 1m), while the other portion evolved into higher-nuclear Pd clusters (Fig. 1n), and no large NPs were observed (Supplementary Fig. 7), which we attribute to Pd anchoring to the more abundant Al^V^ sites on the defective alumina support.

**Fig. 2:**
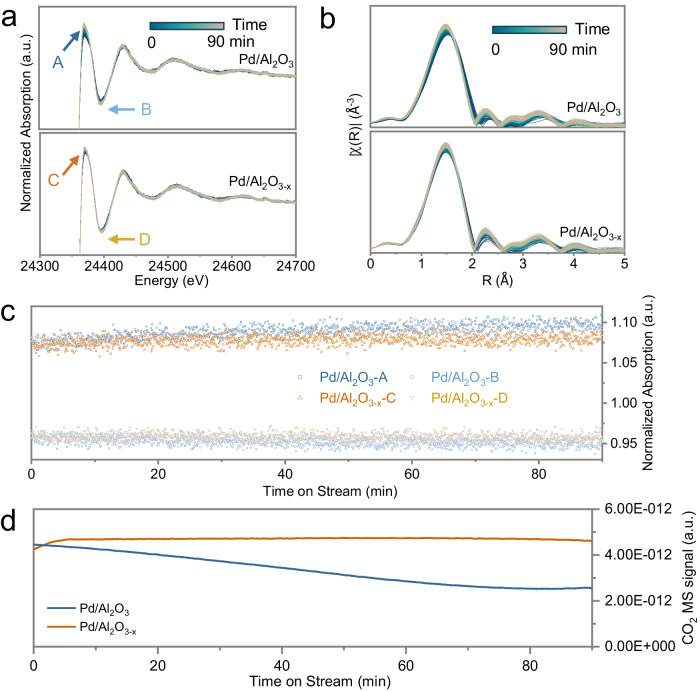
Correlation of Pd redox state and deactivation on different alumina supports. Operando X-ray absorption near-edge spectroscopy (XANES) (**a**) and R-space extended X-ray absorption fine structure (EXAFS) (**b**) of Pd/Al_2_O_3_ (top) and Pd/Al_2_O_3-x_ (bottom) with 5 s time resolution obtained during aging in reaction conditions (CH_4_ : O_2_ : He = 2:8:90, reaction temperature: 500 °C, and GHSV = 9,554 h^−1^). **c** Plot of normalized X-ray absorption at the white line and first minimum (marked in Fig. 2a), as a function of time on stream. The corresponding on-line CO_2_ mass spectrometry (MS) signal collected during the operando X-ray absorption spectroscopy (XAS) measurements (**d**).

In order to study the configuration of Pd species and their transformations during catalytic CH_4_ oxidation, we employed operando QEXAFS with a time resolution of 1s^27–29^. The catalysts were heated in reaction conditions and subsequently tested at 500 °C for 1.5 h, allowing us to track alterations in the Pd structure and compare these with catalyst deactivation. The normalized Pd K-edge X-ray absorption near-edge spectroscopy (XANES) and Fourier transformed EXAFS binned at intervals of 5 s to enhance the signal-to-noise ratio, are shown in Fig. 2a, b. Pd was predominantly in an oxidized state under reaction conditions. As the white line and the first minimum of the XANES are sensitive to a change in structure and oxidation state of the metal species, we plotted the normalized X-ray absorption of the white line (denoted as A for Pd/Al_2_O_3_ and C for Pd/Al_2_O_3-x_) and the first minimum (denoted as B for Pd/Al_2_O_3_ and D for Pd/Al_2_O_3-x_) against time on stream (Fig. 2c). This allowed us to compare the subtle changes in oxidation states of Pd species during the aging process. The white line absorption values of Pd species in Pd/Al_2_O_3-x_ did not change appreciably, while for Pd/Al_2_O_3_ they increased slightly over time, suggesting a change in oxidation state of Pd species during the aging process. The R-space EXAFS indicated that the amount of metallic Pd species (at approximately 2.3 Å) on both catalysts remained roughly unchanged during this process (Fig. 2b), while the Pd-O contribution increased slightly. This can be attributed to growth of PdO NPs, consequently leading to an increase in Pd-O coordination number. It is worthwhile to mention that once steady state is reached under reaction conditions, the energies of the Pd K-edge absorption edge, the structures of the white line peak, and the absence of Pd-Pd scattering in the first shell of the EXAFS, all suggest the absence of metallic Pd as a composition of active centers. The on-line mass spectrometry (MS) results revealed a progressive decline in CO_2_ production over Pd/Al_2_O_3_ in the aging process (Fig. 2d). Consistent with the catalytic test results, these findings suggest a correlation between deactivation and the subsequent oxidation or sintering of Pd species. In contrast, the defective Al_2_O_3-x_ support significantly suppressed the sintering/deactivation of Pd.

### Redox dynamics of Pd clusters and nanoparticles under CH_4_/O_2_ cycles

**Fig. 3:**
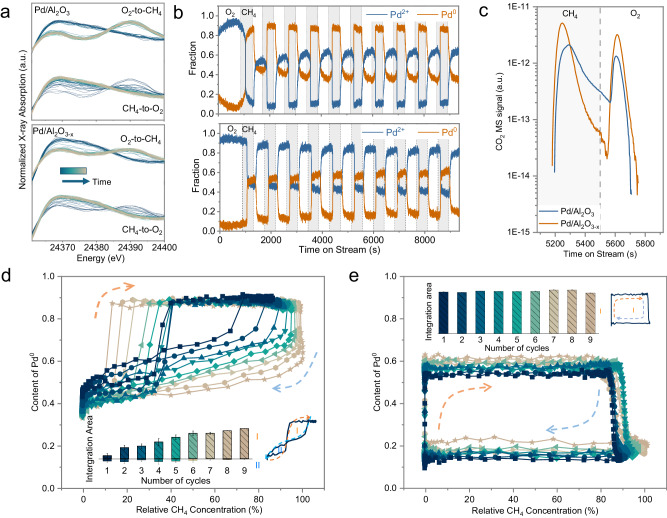
Redox dynamics of palladium species during simulated lean/rich cycles. **a** White line region of operando X-ray absorption near-edge spectroscopy (XANES) recorded at 1 s time resolution obtained upon switching between CH_4_ and O_2_. **b** Plots of Pd^2+^ and Pd^0^ relative contents over Pd/Al_2_O_3_ (top) and Pd/Al_2_O_3-x_ (bottom) catalysts as a function of time, in the alternating CH_4_/O_2_ pulses. (Reaction temperature: 400 °C, and GHSV = 7,643 h^−1^). **c** Plot of online CO_2_ mass spectrometry (MS) signal as a function of time in a representative modulation excitation (ME) cycle. The variation in the content of Pd^0^ species over Pd/Al_2_O_3_ (**d**) and Pd/Al_2_O_3-x_ (**e**) across the first to ninth reduction/oxidation cycles of the ME experiment, as a function of the relative CH_4_/O_2_ composition. The inserted bar chart illustrates the variation in the integrated area of the loops formed by the increase (during the reduction process) and decrease (during the oxidation process) of Pd^0^ species in each oxidation/reduction cycle, as a function of the cycle number. Error bars indicate the 95% confidence interval.

In typical three-way catalytic converters, the exhaust composition undergoes fast perturbations between reducing (also known as fuel-rich) and oxidizing (lean) conditions, close the stoichiometric point^30^. This is known to cause changes in Pd speciation and can cause rapid aging of the catalyst. To mimic such rapid changes in redox conditions and study their effect on Pd species and catalytic activity, we have performed operando QEXAFS with 1 s time-resolution during alternating pulses (i.e., modulation excitation, in short ME) of CH_4_ and O_2_. While the spectra were recorded, the products were measured by online MS at 2.5 s time resolution. (See Supplementary Information, Section 7.4 for details). Time-resolved Pd K-edge XANES of Pd/Al_2_O_3_ and Pd/Al_2_O_3-x_ at the white line region (24360–24400 eV) in a representative ME cycle are shown in Fig. 3a. Because Pd species are considered to follow the Mars-van Krevelen mechanism in CH_4_ oxidation reactions, i.e., the lattice O in PdO may be taken away or replenished in redox reactions, the change in the absorption intensity of the white line can quantitatively describe the change in the average valence of the Pd species^8^. Thus, from the white line feature of the time-resolved XANES, we can infer that the valence of the Pd species on Pd/Al_2_O_3_ changed more significantly than that on Pd/Al_2_O_3-x_ in the ME cycle, and the Pd species on Pd/Al_2_O_3-x_ maintain a higher oxidation state in CH_4_ atmosphere. In the ME experiments, the observed change in Pd species is mainly an interconversion between PdO and metallic Pd. To quantitatively characterize the changes in Pd oxidation state, we performed least squares linear combination fitting (LSLCF) of the Pd XANES, using PdO and metallic Pd as references. Considering that the XANES of atomically dispersed Pd species and PdO are very close (Supplementary Fig. 23), the fitted PdO fraction is the cumulative presence of single-atom Pd, PdO_x_ clusters and NPs. Hence, we designate these components as Pd^2+^. The content of the Pd^2+^ and Pd^0^ species as a function of time in the ME experiment are shown in Fig. 3b. Interestingly, in the initial switch from O_2_ to CH_4_ atmosphere on Pd/Al_2_O_3_, around 90% of the Pd species underwent a transformation from Pd^2+^ to Pd^0^. However, in subsequent periodic pulsations alternating between CH_4_ and O_2_, a proportion of approximately 40% Pd^0^ and 10% Pd^2+^ was consistently maintained, while the remaining 50% of Pd species was oxidized or reduced depending on reaction atmosphere. The presence of irreducible Pd^2+^ species can be attributed to interfacial and single-atom Pd species, while Pd^0^ species that cannot be oxidized may possibly arise from Pd atoms located within the interior of the NPs. These atoms are generated during the initial reduction process but remain metallic throughout the oxidation process probably due to the significant energy barrier associated with the diffusion of O atoms from the surface to the interior^31^.

In the case of Pd/Al_2_O_3-x_, on the other hand, approximately 40% Pd^2+^ and 10% Pd^0^ persisted unaffected by the oxidation/reduction atmosphere. This finding further suggests a higher proportion of interfacial/single-atom Pd species and smaller-sized PdO_x_ clusters/nanoparticles over Pd/Al_2_O_3-x_ compared to Pd/Al_2_O_3_. However, it is worth noting that the total quantity of Pd atoms capable of engaging in oxidation/reduction processes remained relatively similar between the two catalysts, accounting for approximately 50%. We propose that the remainder, non-redox-active species are located at the interface with alumina. Despite their comparable quantity, the redox-active Pd species on Pd/Al_2_O_3_ and Pd/Al_2_O_3-x_ can be anticipated to exhibit different catalytic performance, because of their nature (more/less oxidic and dispersed).

Figure 3c illustrates the plots of the online CO_2_ MS signal as a function of time in a representative ME cycle. Upon switching to a CH_4_ atmosphere, the CO_2_ signal on Pd/Al_2_O_3-x_ exhibited an earlier onset and attained a higher peak compared to Pd/Al_2_O_3_. This potentially indicates a greater reducibility of PdO_x_ clusters than NPs. Furthermore, on Pd/Al_2_O_3-x_ there was a more pronounced decline in the CO_2_ signal subsequent to reaching its peak in comparison with Pd/Al_2_O_3_. This observation may suggest either a lower CO_2_ desorption energy on PdO_x_ clusters, or a higher reduction energy barrier for the sub-surface Pd^2+^ on the PdO_x_ NPs. The latter possibility can be ruled out as it does not align with the reduction rate of Pd^2+^ species indicated in Fig. 3b. Upon switching to the O_2_ atmosphere, CO_2_ MS signals were observed as well, albeit with an integrated area of approximately 30% compared to the previous process. The release of CO_2_ at this stage was attributed to the oxidation of residual CH_4_ from the previous gas pulse and/or to the oxidation of carbonaceous intermediates on the catalyst surface. The integration of CO_2_ MS signals during reduction in CH_4_ and oxidation in O_2_ reveals that the amount of CO_2_ produced on Pd/Al_2_O_3-x_ is 1.45 and 2.92 times higher than on Pd/Al_2_O_3_, respectively. The potential reasons for the higher production of CO_2_ on Pd/Al_2_O_3-x_ can may include: (a) the PdO_x_ clusters are more active than NPs to catalyze CH_4_ oxidation; (b) the higher dispersion of PdO_x_ clusters than NPs allows for the formation of a larger number of carbonaceous intermediates.

Considering gas mixing and reaction during alternating pulses of CH_4_ and O_2_, the ratio of Pd^0^/Pd^2+^ species may exhibit dependence on the gas composition during the formed transients. Time-resolved QEXAFS and MS spectra can be combined in tracking the dependency of Pd species on the gaseous environment in the ME experiment. Since the combined content of Pd^2+^ and Pd^0^, as determined from the unconstrained LCF results, adds up to 1 with less than 0.06 error, our focus is specifically on plotting the changes in the Pd^0^ species content with respect to the relative concentrations of CH_4_ and O_2_ obtained from online MS, to gain a better understanding of the distinct responses of Pd atoms in clusters and NPs to the gas mixture interface during the reduction-oxidation cycles.

The reduction of Pd^2+^ on Pd/Al_2_O_3_ proceeded slowly at lower methane concentrations, necessitating a specific threshold of the CH_4_/O_2_ ratio in the environment to expedite the reduction process. Conversely, during the oxidation process, Pd^0^ were oxidized upon the introduction of a small quantity of O_2_, with the oxidation rate gradually diminishing over time. In a single reduction/oxidation cycle, the reduction and oxidation curves of the Pd species interlace. As depicted in Fig. 3d, we denoted the two intertwined loops formed by the variations in the Pd^0^ species’ content with respect to CH_4_ concentration during the reduction-oxidation cycle as regions I and II. Region I is located at the higher relative CH_4_ concentrations, while region II at the lower relative methane concentrations. A larger area in region I indicates that a greater number of Pd^2+^ species can be reduced at lower CH_4_ concentrations, and more Pd^0^ species can be oxidized at lower relative O_2_ concentrations. Thus, the ratio of the areas I/II can serve as a descriptor of the reactivity of the Pd species. We integrated the areas of regions I and II in each ME cycle, and the changes in these integrated areas with cycle number are displayed in the inset of Fig. 3d. It is worth noting that the integrated area of region I increases with the number of cycles. This suggests that the ME cycling enhances the redox performance of Pd in Pd/Al_2_O_3_.

On the other hand, Pd in Pd/Al_2_O_3-x_ exhibited different responses to the alternating reaction gas pulses in the ME experiment. Specifically, the reducible Pd^2+^ species underwent immediate and complete reduction to Pd^0^ upon contact with CH_4_. Similarly, during the oxidation process, the oxidizable Pd^0^ species rapidly oxidize to Pd^2+^ upon exposure to O_2_. Considering that the relative concentrations of CH_4_ and O_2_ were obtained from online MS, it can be inferred that once Pd species on Pd/Al_2_O_3-x_ were subjected to alternating CH_4_/O_2_ pulses, they preferentially consumed all CH_4_ or O_2_ present in the gas mixture interface through the reduction/oxidation of Pd species, or the catalytic CH_4_ oxidation. This process occurs at such a rapid rate that the apparent composition of Pd species appears to be independent of the relative concentrations of CH_4_/O_2_ in the gas mixture interface. Pd^0^ content as a function of CH_4_ concentration in the CH_4_/O_2_ pulse cycles forms a singular loop, region I in Fig. 3d, serving as a descriptor of the CH_4_ oxidation reactivity. Notably, the integrated area of region III shows minimal sensitivity to the number of ME cycles. As discussed earlier, the reducible/oxidizable Pd species on Pd/Al_2_O_3_ and Pd/Al_2_O_3-x_ originate from non-interface and non-core Pd atoms located on PdO_x_ NPs and PdO_x_ clusters, respectively. The above analysis of their responses to CH_4_ and O_2_ pulses suggests that Pd atoms within PdO_x_ clusters exhibit higher catalytic reactivity towards CH_4_ oxidation compared to PdO_x_ NPs, with a lesser dependence on the relative concentration of CH_4_ or O_2_. With an increasing number of ME cycles, the activity of Pd atoms contained within PdO_x_ NPs gradually improved, although it consistently remained lower than that of PdO_x_ clusters. One possible explanation for this heightened activity is an increase in surface disorder or boundaries^31–33^. The gradual reduction in the amount of non-reducible Pd^2+^ species on Pd/Al_2_O_3-x_ over time indicates the sintering of Pd in the ME experiment. However, the relatively stable area of region I of Fig. 3e implies that sintering does not significantly affect the activity of Pd atoms on the cluster surface.

### Unraveling the reaction mechanism of CH_4_ oxidation on Pd/Al_2_O_3-x_

**Fig. 4:**
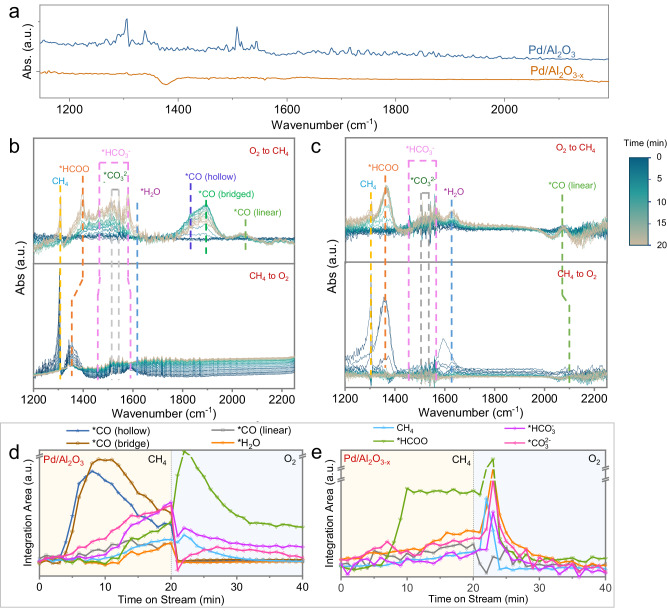
Surface chemistry of palladium clusters and nanoparticles during CH_4_ oxidation. **a** Operando Fourier transform-infrared (FT-IR) spectra of Pd/Al_2_O_3_ (Pd NPs) and Pd/Al_2_O_3-x_ (Pd clusters) at the steady states under reaction condition. CH_4_ : O_2_ : He = 2:8:90, reaction temperature = 400 °C, GHSV = 60,000 mL·g_cat_^−1^·h^−1^. In-situ FT-IR spectra of Pd/Al_2_O_3_ (**b**) and Pd/Al_2_O_3-x_ (**c**) in the successive O_2_-to-CH_4_ and CH_4_-to-O_2_ switching processes, reaction temperature = 400 °C. Plots of the normalized integrated area of the IR absorption of each surface species on Pd/Al_2_O_3_ (**d**) and Pd/Al_2_O_3-x_ (**e**) as a function of time during gas switching experiments.

Operando FT-IR spectroscopy was employed to further investigate the reaction mechanism of catalytic CH_4_ oxidation. In all cases, the FT-IR spectra of the catalysts in a He atmosphere at 400 °C were subtracted (see Supplementary Information, Section 6.1 for details), to visualize the adsorbed organic intermediates. After the aged Pd/Al_2_O_3_ and Pd/Al_2_O_3-x_ catalysts were subjected to reaction conditions (400 °C, CH_4_: O_2_: He = 2:4:90, GHSV = 60,000 h^−1^) the FT-IR spectra were collected until reaching the steady states (Supplementary Information, Section 6.1 and Fig. 4a for details). The assignment of observed FT-IR absorption peaks to organic species is listed in Supplementary Table 6. A higher CH_4_ absorption band at the wavenumber of 1305 cm^-1^ was observed on Pd/Al_2_O_3_ catalyst, because of the lower catalytic activity of this catalyst. Nevertheless, the gradual accumulation of formate, carbonate, and bicarbonate species on the surface of Pd/Al_2_O_3_ was observed. The accumulation of carbonates and bicarbonates as reaction products on Pd/Al_2_O_3_ implies that under reaction conditions, Pd species may be poisoned by these species, leading to reduced activity. This is consistent with the observation of a lower rate of CO_2_ release over Pd/Al_2_O_3_ during ME (Fig. 3c). In contrast, when Pd/Al_2_O_3-x_ is switched into the reaction atmosphere, the catalyst could maintain a relatively cleaner surface, with a certain amount of adsorbed bicarbonate and water, despite higher CH_4_ conversion.

To record the build-up and degradation of the intermediates on the surface after excitation by different reactants, we performed operando FT-IR spectroscopy experiments with a time resolution of 1 min during alternating pulses of CH_4_ and O_2_ at 400 °C. The FT-IR spectra obtained over Pd/Al_2_O_3_ and Pd/Al_2_O_3-x_ are shown in the Fig. 4b, c, respectively. To trace the occupation and exchange of different organic species with time, the FT-IR absorption peaks of all the observed species were integrated (Fig. 4d, e).

When switching from O_2_ to CH_4_ atmosphere, two intermediates, *CO and *HCOO, gradually accumulated on the surface of Pd/Al_2_O_3_ and Pd/Al_2_O_3-x_. The vibrational frequencies of the adsorbed CO on both catalysts are less than 2100 cm^−1^, indicating that surface Pd species are metallic under these conditions^34,35^, which agrees with the operando XAS results (Fig. 3b). Furthermore, it is interesting to note that *CO and *HCOO feature different adsorption modes on Pd/Al_2_O_3_ and Pd/Al_2_O_3-x_, because of their different surface structures. Specifically, the absence of infrared absorption peaks in the wavenumber range of 1800–2000 cm^−1^ (bridge- and hollow- CO absorption bands) on Pd/Al_2_O_3-x_ suggest that the active centers of Pd/Al_2_O_3-x_ are composed of single atoms and small clusters during reaction, because only in that case would geometrical effects prevent the bridge- or hollow-adsorption of CO^36^. Besides, the linearly adsorbed CO on Pd/Al_2_O_3-x_, exhibits a higher vibrational frequency than that on Pd/Al_2_O_3_, implying that the strong interaction between Pd and Al_2_O_3-x_ affords the atomically dispersed Pd species a partial positive charge^37,38^. This is consistent with the observation that the C-H vibration of the adsorbed formate species exhibits a slightly lower frequency on Pd/Al_2_O_3-x_ (1364 cm^−1^) than on Pd/Al_2_O_3_ (1368 cm^−1^)^39,40^. From a time-dependence perspective (Fig. 3c), once switched from O_2_ to CH_4_ atmosphere, the hollow- and bridge-adsorbed CO covers the surface of Pd/Al_2_O_3_ preferentially amongst all intermediates. This can suggest the crucial role of the CO pathway on Pd/Al_2_O_3_. The coverage of the three CO intermediates: hollow-, bridge- and linear-CO, begin to decrease at minutes 8, 11 and 14, respectively. This is either due to the desorption of CO caused by a competition with other adsorbates, or because the CO species are further oxidized by subsurface oxygen. Once the CO intermediates are formed, carbonates begin to accumulate on the surface of Pd/Al_2_O_3_. After ten minutes of CH_4_ flow, when the coverage of carbonate reaches a specific threshold, the infrared absorption peaks for formate, bicarbonate and water become evident on Pd/Al_2_O_3_. Importantly, the accumulation of carbonate and bicarbonate on the surface of Pd NPs may induce alterations in the reaction pathways, favouring the formation of *HCOO. In contrast, although the *CO intermediate on Pd/Al_2_O_3-x_ also appears early in the atmosphere of CH_4_, its coverage remains relatively low. Interestingly, the coverage of *HCOO on Pd/Al_2_O_3-x_ increases rapidly from 6 min onwards and became the dominant surface species.

On switching from CH_4_ to O_2_ atmosphere, *CO are immediately consumed on both Pd/Al_2_O_3_ and Pd/Al_2_O_3-x_, while the coverage of *HCOO increased abruptly. This may indicate that the oxidation of the CO intermediate is kinetically more favorable. More importantly, from the degradation rates of *HCOO we can infer that Pd clusters or single atoms are significantly more active than Pd NPs in catalyzing the oxidation of formate intermediates. As the (OCO)_s_ vibrational frequency of the adsorbed *HCOO is correlated with the oxidation states of the Pd species, it can be deduced that the Pd-O coordination number of the surface Pd over the clusters or single atoms is lower than that over PdO_x_ NPs in the atmosphere of O_2_, because Pd/Al_2_O_3-x_ exhibits a higher *HCOO vibrational frequency (1358 cm^−1^) than Pd/Al_2_O_3_ (1349 cm^−1^). This may account for the higher activity of Pd/Al_2_O_3-x_ towards *HCOO oxidation.

In the previous section, we proposed the hypothesis that Pd single-atoms may also participate in CH_4_ oxidation without yielding Pd^0^ species. That is, the Pd single atoms may accomplish a catalytic cycle through a combination of oxidative addition, reductive elimination, and transfer insertion, which is similar to the extensively reported catalytic mechanism of a homogeneous catalyst^41,42^. In light of the difficulty to corroborate this hypothesis experimentally, ab initio DFT calculations may afford us an insight into CH_4_ oxidation pathways over the Pd single atoms.

**Fig. 5:**
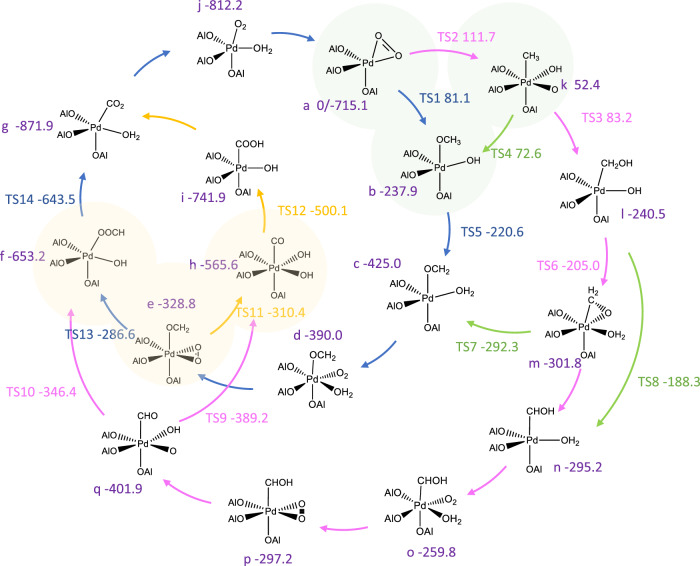
A model of CH_4_ oxidation pathways on single-atom Pd. Reaction pathway network for CH_4_ oxidation on single-atom Pd obtained from density functional theory (DFT) calculations (top). The values are the corresponding Gibbs free energies of the intermediates and transition states, in kJ/mol. The blue, yellow, pink, and green arrows respectively represent the formate branch of *OCH_3_ pathway, the *CO branch of the *OCH_3_ pathway, the *OCH_2_ branch of the *CH_3_ pathway, and the interconversions between these pathways. The green shaded region represents the elementary steps for CH_4_ activation, leading to either *CH_3_ or *OCH_3_ pathways; while the yellow shaded region is the elemental steps where branching occurs between *CO and formate paths on the *OCH_3_ pathway.

**Fig. 6:**
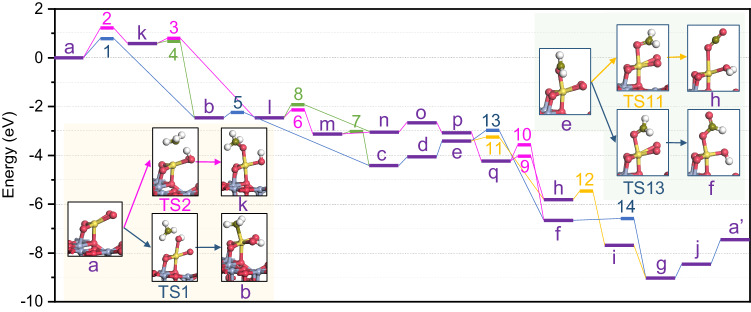
Gibbs free-energy profile of CH_4_ oxidation on single-atom Pd. The letters and numbers annotated on the energy profile represent the intermediates and transition states along the various reaction pathways, corresponding to those depicted in Fig. 5. The green shaded region represents the elementary steps for CH_4_ activation, leading to either *CH_3_ or *OCH_3_ pathways; while the yellow shaded region is the elemental steps where branching occurs between *CO and formate paths on the *OCH_3_ pathway.

In spite of the considerable theoretical effort being expended on the mechanism of the CH_4_ oxidation over the specific facets of Pd or PdO, comparable work has rarely been done on the single-atom Pd species^32,43–49^. To implement this knowledge and examine our hypothesis, we have built a Pd_1_/Al_2_O_3_ slab in which an isolated Pd atom was anchored on the (100) facet of γ-Al_2_O_3_ via O bridges^19^ (Supplementary Information, Section 8.1 for details). Referring to the individual CH_4_ oxidation mechanisms postulated by previous studies, we proposed all CH_4_ oxidation pathways over a single-atom Pd, and subsequently screened and optimized these pathways with DFT calculations. The proposed mechanism network is illustrated in Fig. 5, where a-j represent sketches of the structures of the catalyst (a) and different intermediates (b-j). The numbers following the letters are the calculated Gibbs free energies of the corresponding intermediates relative to the initial catalyst, at 400 °C. The arrows symbolize the conversion between individual intermediates, and TS1-14 refer to the transition states present in these conversion processes, whose relative Gibbs free energies are also indicated below. The configuration of the individual intermediates and transition state species is shown in Supplementary information, Sections 8.2 and 8.4. For comparison purposes, an energy profile of the entire reaction network was made (Fig. 6).

It is worth noting that the well-known carbide pathway is highly unlikely on the single-atom Pd, as the adsorption of carbene, carbyl and *C species on the single-atom Pd is thermodynamically unfavorable (Supplementary information, Section 8.2 for details). The activation of CH_4_, highlighted in green shading in Fig. 5, is identified as a crucial rate-determining step in the overall CH_4_ oxidation reaction. Compared to the *CH_3_ pathway (marked by pink arrows), the *OCH_x_ pathway (marked by blue arrows) is more favorable in terms of methane activation, as the *OCH_x_ generation has a significantly lower activation energy than the *CH_3_ production. The subsequent oxidation of the carbonous intermediates all exhibits small energy barriers regardless of the pathways. The interconversions (marked by the green arrows) amongst the two pathways are all in favor of OCH_x_ pathways from the microkinetic perspective. These DFT results are corroborating the dominance of the OCH_x_ pathway in the catalytic mechanism.

According to the proposed model, the *CO and *HCOO intermediates observed in the operando FT-IR spectra may have originated from the oxidation of *OCH_2_ by the coexistent active O (highlighted with yellow shadowing). Compared to *CO, the generation of *HCOO is thermodynamically more favorable while kinetically unfavorable, but the energy barrier difference (23.8 kJ/mol) between these two intermediates is much smaller than the thermodynamic Gibbs free energy difference (87.6 kJ/mol). This may explain the preferential appearance of *HCOO adsorbates. Notably, possibly because a reductive elimination leads to large changes in the valence of the single atom Pd, a high energy barrier for the desorption of water from the single-atom Pd was observed, which was higher than the activation energy of the CH_4_ molecule. This is also consistent with the operando FT-IR spectroscopy results, implying that water can poison the single-atom Pd centers.

In comparison with the reaction pathways over a crystalline PdO surface unveiled by previous research^43^, single-atom Pd manifests 20.3 kJ/mol higher CH_4_ activation energy but lower energy barriers for further oxidation of carbon-containing intermediates. This explains the relatively cleaner surface of Pd/Al_2_O_3-x_ under the reaction conditions as shown in the operando FT-IR spectra. The other rate-determining step to the single-atom Pd, the desorption of water, is less demanding for the crystalline PdO thermodynamically^43^. From a practical point of view, the low tolerance of Pd single atoms to water vapor, just as the poor resistance of PdO_x_ NPs to carbonates, may be detrimental to their activity, regardless of their high dispersion or intrinsic activity. These are conceivably important reasons for the growth of apparent activation energy during the deactivation of commercial Pd/Al_2_O_3_ catalysts and highlight the potential of Pd clusters as more stable active centers for CH_4_ activation.

To sum up, a highly dispersed Pd catalyst was synthesized using defective Al_2_O_3-x_ as a support and exhibited higher catalytic activity and stability in CH_4_ oxidation compared to a counterpart Pd/Al_2_O_3_ catalyst. The evolution of the Pd active centers was significantly influenced by the type of support under the reaction conditions: the Pd clusters on γ-Al_2_O_3_ partially sintered and agglomerated into PdO_x_ nanoparticles (NPs), meanwhile being partially anchored as single atoms. In contrast, only a small fraction of the initial Pd single atoms in Pd/Al_2_O_3-x_ were fused into clusters due to the constraint effect of Al^v^ species. The evolution of active centers under reaction conditions not only changes the quantity of Pd atoms available for participating in catalytic reaction, but also alters the intrinsic activities of the catalysts and their resistance to different poisoning species by changing the relative proportions of Pd NPs, clusters, and single atoms. The combined operando FT-IR spectroscopy, operando QEXAFS and DFT calculations revealed the mechanism of CH_4_ oxidation reactions over Pd NPs, clusters and single atoms. The higher dispersion and lower adsorbate coverage of PdO_x_ clusters made them the most active in catalytic CH_4_ oxidation. This paper showcases a method to ensure the abundance of Pd clusters by imposing restrictions on the evolution of Pd with defective alumina. We foresee that similar methods may be applied to other supports and metals, paving the way to more thermally stable and highly dispersed metal catalysts.

## Methods

### Catalyst synthesis

The Al_2_O_3-x_ support was synthesized by calcining an eutectoid composed of urea (CON_2_H_4_, AR, Sigma-Aldrich), thiourea (CSN_2_H_4_, AR, Sigma-Aldrich) and aluminum sulfate octadecahydrate (Al_2_(SO_4_)_3_·18H_2_O, AR, Sigma-Aldrich) in a tubular oven at 550 °C for 4 h. Parameters, such as feedstock types, feedstock ratio, calcination temperature and heat treatment time, were individually optimized to obtain the highest Al^V^ content, which are discussed in Supplementary information. The incorporation of 1 wt% of Pd onto γ-Al_2_O_3_ and Al_2_O_3-x_ was approached by the incipient wetness impregnation (IWI) method, using palladium (II) chloride (PdCl_2_, AR, Sigma-Aldrich) in methanol (AR, Sigma-Aldrich) as precursor, followed by heat treatment at 400 °C in air for 2 h (Supplementary information for further details).

### Catalyst characterization

The crystalline structures, porous properties, morphology, and Al coordination structures of the support and catalysts were characterized using powder X-ray diffraction (XRD), gas physisorption measurements at 77 K, transmission electron microscopy (TEM), and solid-state ^27^Al nuclear magnetic resonance spectroscopy (NMR), respectively. The presence of Pd were characterized by high-angle annular dark-field scanning transmission electron microscopy (HAADF-STEM) and Fourier transform infrared (FT-IR) spectroscopy of adsorbed CO. Full details are provided in the Supplementary information.

### Catalyst testing

The catalysts were evaluated for CH_4_ oxidation experiments in a quartz tube (inner diameter = 8 mm) reactor with a down flow over 75–212 μm catalyst particles, diluted by SiC crystals to make the catalyst bed volume up to 500 µL. The volume composition of the feed gas was 2 vol.% CH_4_, 8 vol.% O_2_ balanced with high purity helium. Stability tests and temperature-resolved activity tests were performed at gas hourly space velocities (GHSV) of 120,000 and 60,000 h^−1^, respectively. On-line product analysis was performed with an Interscience custom-built Global Analyzer Solutions Compact GC4.0 gas chromatograph (GC). Blank experiments showed that reaction rates were negligible without catalyst.

### Operando quick-XAS with on-line product analysis

Operando quick-X-ray absorption spectroscopy (Q-XAS) experiments with 1-s time resolution were performed at the SuperXAS beamline (X10DA) at the Swiss Light Source (SLS) in fluorescence mode. The X-ray beam from the 2.9 T bending magnet was collimated by a Pt coated mirror and monochromatized with a Si(111) channel-cut crystal Quick-XAS monochromator. The Si(111) crystal was rotated at a frequency of 1 Hz across the Pd K-edge, and the signals of the 20 cm long Ar/N_2_ filled ionization chambers, a PIPS diode and the angular encoder were sampled at a frequency of 2 MHz. The edge energy for the Pd spectra was calibrated using a Pd foil in transmission mode, which was collected simultaneously with the quick-extended X-ray absorption fine structure (Q-EXAFS) spectra of the sample. The measurements were performed in a custom-built operando reaction cell (Supplementary information 7.1). Q-XAS data was subsequently evaluated using the ProQEXAFS software as well as self-developed Matlab™ code. Further information about the Q-XAS data processing can be found in the Supplementary information.

### Operando FT-IR spectroscopy with on-line product analysis

Operando Fourier transform infrared (FT-IR) spectroscopy measurements with 1 min time resolution were carried out on a Bruker Tensor 37 FT-IR spectrometer equipped with a DTGS detector, to study the time-resolved formation and decomposition of the intermediates in CH_4_ oxidation over different Pd species, under different reaction conditions. Product formation was followed by an Interscience custom-built Global Analyzer Solutions Compact GC4.0 gas chromatograph (GC). More experimental details, such as the design of the operando FT-IR cell, the preparation of the catalyst pellets, the experimental procedure and reaction conditions of the operando FT-IR spectroscopy measurements, and the data collection and processing methods can be found in the Supplementary information.

### Density functional theory calculations

Ab initio density functional theory (DFT) calculations were performed using the Vienna Ab-initio Simulation Package (VASP)^50^ with the projector-augmented wave (PAW) method^51,52^. The Perdew-Becke Ernzerhof (PBE) exchange-correlation functional was used^45^. The (100) facet of γ-Al_2_O_3_ obtained from Digne et al. was optimized and applied as the support^53,54^, because the abundant Al^V^ sites on this facet^19^. A height of 15 Å in the z direction was used to separate the surface slab, in order to prevent interaction of intermediates. Isolated Pd atom was anchored to Al^V^ sites via oxygen bridges and the most stable position of Pd is tested on the Al_2_O_3_ (100) slab (see Supplementary information). All atoms were allowed to relax. The kinetic energy cutoff for the plane wave basis set was 500 eV. The Monkhorst-Pack mesh k-points of (5 × 3 × 1) for Pd_1_/Al_2_O_3_ and Al_2_O_3_ slab were used to sample the surface Brilliouin zone to assure accuracy. Structural optimization was performed by use of the conjugate gradient method, the geometries were converged to 10^−4^ eV and electronic convergence was set at 10^−5^ eV. For the gas-phase calculations, the CH_4_, CO_2_, O_2_, and H_2_O molecules were placed at the center of a 10 × 10 × 10 Å unit cell, with only a G cantered grid for k-point sampling. A width of 0.00002 eV was applied for electron smearing. The reaction pathways discussed in the main text have been calculated by the nudged elastic band (NEB) approach as implemented in VASP^54,55^. To confirm that all transition geometries were in a first-order saddle point on the potential energy surface, the frequency analysis was performed. The Hessian matrix was constructed using a finite displacement approach with a step size of 0.02 Å for displacement of individual atoms along each Cartesian coordinate. These frequencies were used to determine the zero-point energy (ZPE) correction to the energy of the geometries of the initial, transition, and final states.

### Supplementary information

Supporting information

Peer Review File

## Data Availability

All data utilized in the manuscript have been uploaded to the YODA repository and are available under https://science.yoda.uu.nl/research/browse?dir=%2Fresearch-published-papers%2FYu%2C%20X_NatureCommunications_2024.
