# Supplementary material for: Anchoring PdOx clusters on defective alumina for improved catalytic methane oxidation
Source: Nat Commun. 2024 Aug 1;15:6494. doi: 10.1038/s41467-024-50216-0 (PMC11294617; doi:10.1038/s41467-024-50216-0)
Supplement: Supplementary file 1 — Supporting information [file 41467_2024_50216_MOESM1_ESM.pdf]

Supplementary information

Anchoring PdO_x Clusters on Defective Alumina for Improved Catalytic Methane Oxidation

Xiang Yu[1], Nina S. Genz[1,2], Rafael G. Mendes[3,4], Xinwei Ye[1], Florian Meirer[1], Maarten Nachtegaal[2], Matteo Monai[1,*], and Bert M. Weckhuysen[1,*]

Affiliations:

[1] Inorganic Chemistry and Catalysis, Debye Institute for Nanomaterials Science & Institute for Sustainable and Circular Chemistry, Utrecht University, Universiteitsweg 99, 3584 CG Utrecht, The Netherlands

[2] Paul Scherrer Institute, Forschungsstrasse 111, 5232 Villigen, Switzerland

[3] Soft Condensed Matter, Debye Institute for Nanomaterials Science, Utrecht University, Princetonplein 5, 3584 CC Utrecht, The Netherlands

[4] Interfaces, Confinement, Matériaux et Nanostructures, CNRS-Orléans, 1b rue de la Férollerie, 45071 Orléans, France

Corresponding authors: m.monai@uu.nl, b.m.weckhuysen@uu.nl

Supplementary methods

Materials

Urea (CON_2H_4), thiourea (CSN_2H_4), methanol (CH_3OH), aluminium sulfate octadecahydrate ($Al_2(SO_4)_3 \cdot 18H_2O$), aluminium nitrate nonahydrate ($Al(NO_3)_3 \cdot 9H_2O$), aluminum chloride ($AlCl_3$) and palladium (II) acetylacetonate ($C_{10}H_{14}O_4Pd$) were purchased from Sigma-Aldrich. All the above chemicals were of analytical grade and used as purchased without any further purification. γ-Al_2O_3 support was obtained from Engelhard. The deionized (DI) H_2O used in the work had a resistivity of 18.23 mΩ.

Synthesis of Al_2O_{3-x}

3.0 gram of urea, 2.4 gram of thiourea, and 3.0 g of aluminium sulfate octadecahydrate ($Al_2(SO_4)_3 \cdot 18H_2O$) were mixed under continuous grinding for 10 min. Next, the mixture was placed in a 10 mL quartz boat with an aluminum foil as the cover, and then heated to 120 °C for 30 min and subsequently to 550 °C for 2 hours at the ramp of 5 °C /min in tubular oven in air. That resultant solid was collected and thoroughly ground to form a fine powder (<72 μm).

Using the same synthesis conditions, when the quantity of $Al_2(SO_4)_3 \cdot 18H_2O$ were set to 1.8, 3.0, 4.2, and 5.4 g, the resultant solid after calcination were noted as 3$Al_2(SO_4)_3$-5U4T, 5$Al_2(SO_4)_3$-5U4T, 7$Al_2(SO_4)_3$-5U4T, and 9$Al_2(SO_4)_3$-5U4T, respectively.

Likewise, using the same synthesis procedure, but a calcination temperature of 450, 550, and 650 °C, we prepared 5$Al_2(SO_4)_3$-5U4T-450, 5$Al_2(SO_4)_3$-5U4T-550, and 5$Al_2(SO_4)_3$-5U4T-650, respectively.

Using the same synthesis procedure, when the calcination time was set as 1, 2, and 4 h, the obtained materials were denoted as 5$Al_2(SO_4)_3$-5U4T-1h, 5$Al_2(SO_4)_3$-5U4T-2h, and 5$Al_2(SO_4)_3$-5U4T-4h respectively.

With the same synthesis procedures, when the aluminum precursors were replaced by γ-Al_2O_3, $AlCl_3$, and $Al(NO_3)_3$ $9H_2O$, the resulting supports are noted as Al_2O_3-5U4T, $AlCl_3$-5U4T, and $Al(NO_3)_3$-5U4T, respectively. For comparison in this term, the Al_2O_{3-x} support with $Al_2(SO_4)_3 \cdot 18H_2O$ as the aluminum precursor are also noted as $Al_2(SO_4)_3$-5U4T.

Synthesis of Pd/Al_2O_{3-x} and Pd/Al_2O_3

The 5$Al_2(SO_4)_3$-5U4T-4h support will be denoted as Al_2O_{3-x} in the following sections. For the synthesis of Pd/Al_2O_{3-x}, we employed a twice-incipient impregnation-oxidation method to incorporate Pd onto supports. Specifically, 1.43 g of palladium (II) chloride was dissolved into methanol (100 mL) to form a solution. Next, 500 μL of as-prepared solution were added into Al_2O_{3-x} (500 mg), followed by vigorous stirring to form a thick slurry. After the slurry was evaporated at room temperature for 12 h and subsequently at 100 °C for 5 h, the impregnation-dry process was repeated on the obtain solid once. Afterwards, the yellow powder was oxidized in a muffle oven at 400 °C for 2 h, with a ramp of 5 °C/min.

The nominal composition is 1.0 wt.% Pd for the component. Likewise, Pd/Al_2O_3 catalyst was synthesized using γ-Al_2O_3 as the support.

Ex-situ Characterization methods

Powder X-ray diffraction (XRD) was performed on an X'Pert Pro automatic powder diffractometer operated at 35 kV and 15 mA using CuKa (k = 0.15406 nm) monochromatized radiation in all cases, The spectra were collected in the 2-theta range of 10–90° with an angular step of 0.05° and a counting time of 2 s per step. The specific surface areas of the samples were measured by Ar adsorption-desorption at 77 K on Micromeritics Tristar II 3020 Surface Area Analyzer. The samples were first degassed in vacuum at 200 °C for 2 h before the measurements. Specific surface areas were calculated using Brunauer-Emmett-Teller (BET) method in the relative pressure (P/P_0) range of 0.05–0.25. The pore size distribution of samples was calculated through the Barrett-Joyner-Halenda (BJH) methods. The pore sizes were obtained from the peak positions of distribution curves. Total pore volume was accumulated at relative pressure of P/P_0=0.99. The organic elemental analysis carried out on the Vario ELIII elemental analyzer (German company). Inductively coupled plasma-optical emission spectrometry (ICP-OES) was conducted over Horiba Ultra II, equipped with photomultiplier tube. TEM studies were performed on a Tecnai20F transmission electron microscope operating at 200 kV. The high-resolution STEM-HAADF images were acquired using a Thermo Fisher Scientific Spectra300 TEM operating with an acceleration voltage of 300 kV, camera length 91 mm, convergence angle 25 mrad, and an acquisition dwell time of 2 ms/pixel. The 27Al MAS NMR measurements were carried out on a Varian 300 MHz NMR spectrometer using a 7.5 mm HX MAS probe with a spinning rate of 12 kHz at resonance frequencies of 156 MHz.

CO-adsorption FT-IR spectroscopy was performed on a PerkinElmer System 2000 instrument in a transmission mode (64 scans, 4 cm^{-1} resolution, DTGS detector, cell with KBr windows). The catalyst powders were pressed into wafers of approximately 16 mm in diameter, and around 0.1 mm thickness weighing between 20–25 mg. These self-supported catalyst wafers were created using a Specac Laboratory Pellet Press, a diaphragm vacuum pump and around 3 t of pressure. To investigate the state of Pd on Pd/Al_2O_3 and Pd/Al_2O_{3-x} with CO-probe molecules, the catalysts wafers were mounted on a stainless-steel cell followed by reducing in a flowing H_2 at 300 °C and ambient pressure in situ. Prior to the test, the catalyst was evacuated to less than 0.5 mbar to remove residual H_2 and water. Thereafter, the cell was cooled with liquid N_2 temperature down to 90 K, and a mixture of 10 % CO/He v/v (Linde AG, 99.999%) was dosed stepwise into the cell via a stainless-steel manifold and a valve to 1 mbar pressure. The IR spectra of the catalysts measured at the same temperature and pressure but in an atmosphere of N_2, were subtracted to obtain the IR spectra of the adsorbed CO molecules.

Supplementary Tables

Supplementary Table 1. Organic elemental analysis of Al_2O_{3-x} calcined at 550°C for 4 h

Sample	C	N	S	H
Al_2O_{3-x}	-	0.02 wt.%	-	1.23 wt.%

Supplementary Note 1: The organic elemental analysis shown in Tables 1 did not detect residual C and S on the Al_2O_{3-x} after calcination at 550°C for 4 h, while some traces of N were observed. This indicates that the organic additive and sulfate anion were removed under the synthesis conditions.

Supplementary Table 2. Physicochemical properties of Al_2O_{3-x} and Al_2O_3 support measured by Ar sorption isotherms.

Sample	BET surface area ($m•g^{-1}$)	Pore volume (cm^3g^{-1})	Average pore size (nm)
Al_2O_{3-x}	420.1	1.0	14.3
Al_2O_3	197.8	0.5	9.9

Supplementary Note 2: The specific surface area, pore volume and average pore size were calculated using BET and BJH method, respectively. The results are shown in Tables 2. It is noteworthy that as-synthesized alumina support has a high specific surface area, reaching 420.1 m^2g^{-1}. Its high specific surface area may originate from the gases released by the decomposition of urea and thiourea during calcination, which formed a large amount of micropores inside the Al_2O_{3-x} nanoparticles (NPs). In addition, Al_2O_{3-x} also possessed a high pore volume (1.0 cm^3g^{-1}) and a large mesopore size (14.3 nm). The hierarchical micro-mesopore structure can be expected to be beneficial for the diffusion during catalytic reactions.

Supplementary Table 3. Physicochemical properties of Al_2O_{3-x} support measured by Ar sorption isotherms.

Sample	BET surface area ($m \cdot g^{-1}$)	Pore volume (cm^3g^{-1})	Average pore size (nm)
Pd/Al_2O_{3-x}	197.3	0.4	30.4
Pd/Al_2O_3	186.9	0.5	10.8

Supplementary Table 4. Comparison of catalytic methane oxidation activities with recent studies

Catalysts	CH_4 Concentration (%)	O_2 Concentration (%)	Space velocity (ml•g^{-1}•h^{-1})	T50 (°C)	reference
1Pd/Ceria-800°	0.5	2.5	60 000	420	[1]
Al-Pd/MA-F	0.5	10	40 000	330	[2]
Pd/Na-MOR	1	4	70 000	325	[3]
Pd_1/CeO_2	0.068	14	300 000	280	[4]
$1Pd/2Pt@CeO_2$	0.068	14	300 000	320	[5]
Pd/NA-Al_2O_3-1000	1	20	15 000	280	[6]
Pd/Al_2O_{3-x}	2	8	60 000	320	This work

T50: The reaction temperature at which the catalytic methane conversion reaches 50%.

Supplementary Note 3: The catalytic activity of Pd/Al_2O_{3-x} after reaching steady state is compared with recently reported Pd catalysts, shown in Supplementary Table 4. The reaction temperature required for achieving a 50% methane conversion is used as a parameter for comparison. Despite differences in reaction setups and conditions, apparently, it can be concluded that Pd/Al_2O_{3-x} still exhibits one of the most competitive catalytic activities.

Supplementary Table 5. The catalytic reaction program of the operando FT-IR spectroscopy experiments.

Step	Time (min)	Ramp (°C/min)	Target Temperature (°C)	He flows (mL/min)	CH_4 flows (mL/min)	O_2 flows (mL/min)
I	0	5	500	25	0	0
II	96	0	500	25	0	0
III	126	0	500	22.5	0.5	2
IV	216	5	400	25	0	0
V	236	0	400	25	0	0
VI	256	0	400	22.5	0.5	2
VII	276	0	400	23	0	2
VIII	296	0	400	24.5	0.5	0
IX	316	0	400	23	0	2
X	336	0	400	22.5	0.5	2
XI	356	20	20	25	0	0

Supplementary Table 6. Assignment of the FT-IR peaks used in this work[7-9].

Species	Absorption band (cm^{-1})					
	Pd/Al_2O_3			Pd/Al_2O_{3-x}		
	CH_4	O_2	Rct	CH_4	O_2	Rct
*CO	2053	-	-	2075	2104	-
	1895	-	-	-	-	-
	1832	-	-	-	-	-
*HCOO	1397	1349	1353	1364	-	-
$*CO_3^{2-}$	1509	1507	1504	1503	-	-
$*HCO_3^-$	1542	1543	1538	1536	-	-
	1496	1457	1459	1458	-	1477
	1580	1588	1598	1568	-	1583
H_2O	1618	-	1621	1630	-	1629

CH_4, O_2, and Rct (reaction feedstock mixture) represent the atmospheres under which the spectra were acquired.

Supplementary Table 7. Linear-combination fitting results of the XANES.

Component	Linear combination content		R-factor	Red. chi-square
	Pd^{2+}	Pd^{0}		
Component A-Pd/Al_2O_3	0.973	0.027	0.03625	0.00479
Component B-Pd/Al_2O_3	0.015	0.985	0.00861	0.00091
Component A-Pd/Al_2O_{3-x}	0.985	0.015	$9.27e^{-5}$	$1.24e^{-5}$
Component B-Pd/Al_2O_{3-x}	0.335	0.665	0.0024	0.00034

Supplementary Table 8. The adsorption energies of PdO_2 on the different site of Al_2O_3 (1 0 0)

Sites	Adsorption Energy (eV)
1&2	-1.558
2&3	-0.772
3&4	-0.728

Supplementary Table 9. The Gibbs free energy of the intermediates adsorbed on the single slab or the corresponding supercells.

Slab	Gibbs Free Energy (kJ/mol)
c	-425.0
c2	-426.3
f	-653.2
f2	-654.6
h	-565.6
h2	-568.4
k	52.4
k2	51.4
n	-295.2
n2	-294.9
q	-401.9
q2	-401.9

Supplementary Figures

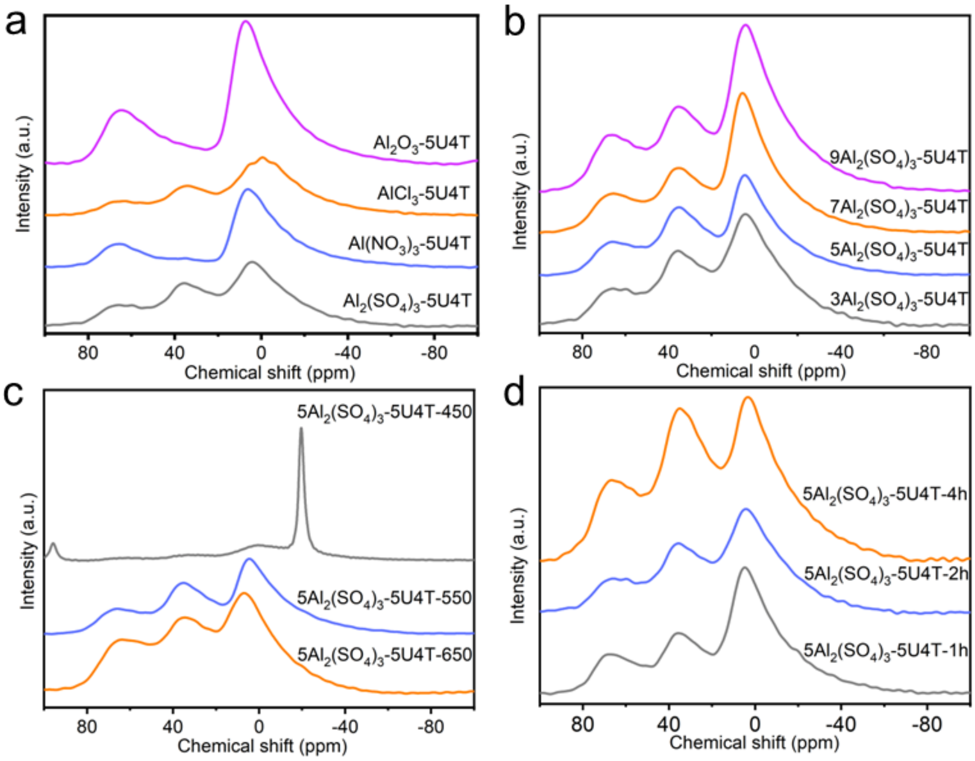

Supplementary Figure 1. The ^{27}Al SSNMR spectra of Al_2O_{3-x}, synthesized under different conditions: **a.** different aluminum precursors type; **b.** different $Al_2(SO_4)_3 \cdot 18H_2O$ contents; **c.** different calcination temperature; and **d.** different calcination time.

Supplementary Note 4: Although penta-coordinated alumina (Al^V) is important for immobilizing the atomically dispersed metal species[10-12], the synthesis of Al^V-rich alumina support was so far elusive because of the strong chemical bonding between Al and oxygen[13-14]. Past studies have shown that the bottom-up approach is possible when some organic molecules are employed as inducers in the synthesis of Al_2O_{3-x}[11, 15]. Nevertheless, the use of such expensive organic molecules poses a barrier to large scale industrial application. We discovered by serendipity that aluminum salts can be dissolved easily in a cheap, deep eutectic solvent (DES) consisting of urea and thiourea, at 80 °C. For $Al_2(SO_4)_3 \cdot 18H_2O$, the solubility can exceed 150 g/100 g of solvent at 80 °C. We conjecture that the solvation of Al cations after the removal of bound water from $Al_2(SO_4)_3 \cdot 18H_2O$ at 120 °C, allows the ligand exchange between sulfate and urea or thiourea. Despite the thermodynamic decomposition temperature of aluminum sulfate exceeding 770 °C, in the DES the solvated Al cations and sulfate anions are effectively isolated from each other. This ensures that the decomposition of sulfate can occur at relatively lower temperatures.[16] Urea or thiourea as carbon nitride precursors possess a high decomposition temperature because of their polymerization during calcination. Therefore, the solvated Al cations will be protected by urea and

thiourea, forming alumina at higher temperatures. The decomposition of urea and thiourea, which are directly coordinated with Al, would allow the creation of oxygen vacancies on alumina, in the meantime generating a large number of pores. Therefore, we deduced that calcination of aluminum salts dissolved in DES would be an effective strategy to synthesize an Al^{V}-rich Al_2O_{3-x} support.

Solid-state magic-angle spinning nuclear magnetic resonance spectroscopy (MAS NMR) has proven to be a powerful tool to differentiate and quantitatively study the content of Al on alumina with different coordination structures[17]. In the ^{27}Al MAS NMR spectra of Al_2O_{3-x} (Scheme 1), three prominent peaks at 7, 38 and 70 ppm are observed, which can be ascribed to Al^{3+} species in tetrahedral (AlO_4 at 7–10 ppm), pentahedral (AlO_5 at 38 ppm) and octahedral (AlO_6 at 70 ppm) coordination, respectively.[10, 17-18] To optimize the synthesis so as to create a maximum Al^{V} content on the support, we investigated the effect of aluminum precursor type, Al precursor content, calcination temperature and calcination time on the ^{27}Al MAS NMR spectra. According to the results shown in Supplementary Figure 1a, all the Al^{3+} salts ($AlCl_3$, $Al(NO_3)_3$, $Al_2(SO_4)_3$) could be employed as the precursors of Al_2O_{3-x} in spite of their different levels of promotion to the formation of Al^{V}, while Al_2O_3 maintained stable in the synthesis procedures, because it could not dissolve in the DES. Specifically, aluminum sulfate is probably the best candidate because of the high Al^{V} yield. By the same token, from Supplementary Figure 1b-1d we can conclude that a medium $Al_2(SO_4)_3 \cdot 18H_2O$ salt content (3.0 g), moderate calcination temperature (550 °C) and long calcination time (4 hours) is preferred to acquire more of Al^{V} centers. In addition, it is worth noting that a sharp peak at -23 ppm was observed in the ^{27}Al MAS NMR spectra (Supplementary Figure 1c) when the calcination temperature is as low as 450 °C, suggestion the presence of a certain amount of $Al_2(SO_4)_3$ residues in the solid products. This reminds of the necessity of high calcination temperatures for sulfate removal.

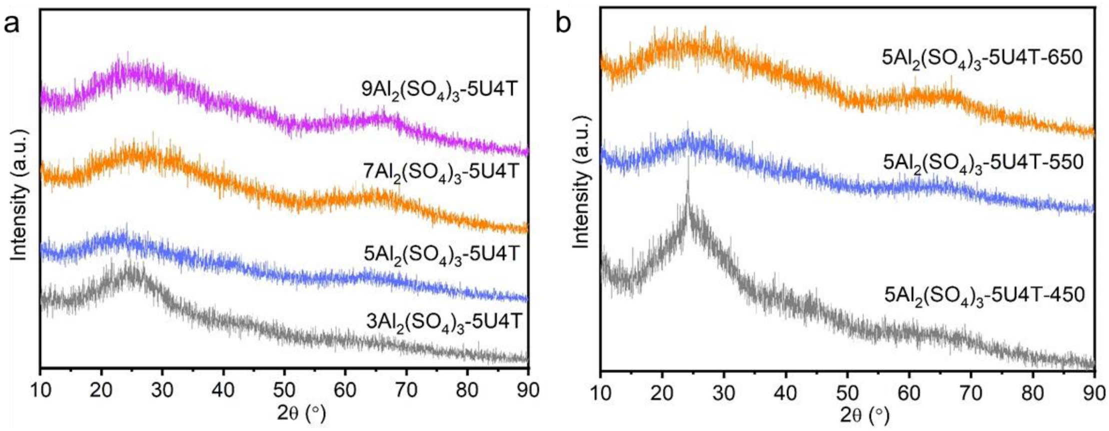

Supplementary Figure 2. XRD patterns of Al_2O_{3-x} synthesized under different condition: **a.** different amount of $Al_2(SO_4)_3 \cdot 18H_2O$ and **b.** different calcination temperature.

Supplementary Note 5: The effect of synthesis conditions, especially the amount of $Al_2(SO_4)_3 \cdot 18H_2O$ and calcination temperature, on the crystal structure of Al_2O_{3-x} was studied with XRD. As shown in Supplementary Figure 2a and 2b, the generated Al_2O_{3-x} are amorphous. Especially at smaller $Al_2(SO_4)_3 \cdot 18H_2O$ contents, the produced Al_2O_{3-x} is highly amorphous (Supplementary Figure 2a). With increasing $Al_2(SO_4)_3 \cdot 18H_2O$ content, the diffractions at a 2θ of 43° and 67° gradually increases, which are the characteristic diffractions of γ-Al_2O_3 (JCPDS 04-0880). It should be noted that despite the appearance of these diffractions, their large diffraction peak widths indicate that γ-Al_2O_3 only appears as localized, short-term ordered crystals. It also demonstrates the preference of the formation of γ-Al_2O_3 at this calcination temperature, comparing with other Al_2O_3 phases. Similarly, from Supplementary Figure 2b we can infer that higher calcination temperatures will drive the transition of Al_2O_{3-x} from amorphous to γ-Al_2O_3 phase. Furthermore, when the calcination temperature was set to 450 °C, a sharp X-ray diffraction peaks at the 2θ of 24° was found, which was attributed to $Al_2SO_4(OH)_4 \cdot 7H_2O$ (JCPDS 43-0669). The presence of residues of sulfate anions at this calcination temperature are in agreement with the aforementioned NMR results (Figure 1c).

Since we confirmed that the alumina obtained from the calcination of the mixture of DES with 3.0 g of $Al_2(SO_4)_3 \cdot 18H_2O$ at 550 °C for 4 hours, possessed the highest Al^V content, which reached a 40 mol.% according to the integration of the NMR spectra (Fig. 1b in the main text). While higher Al^V content might be achieved by tuning the synthesis conditions, we deemed this to be sufficient for anchoring metal cations as a support. Therefore, the alumina synthesized under this condition was used as the support and will be referred to as Al_2O_{3-x} in the following discussion.

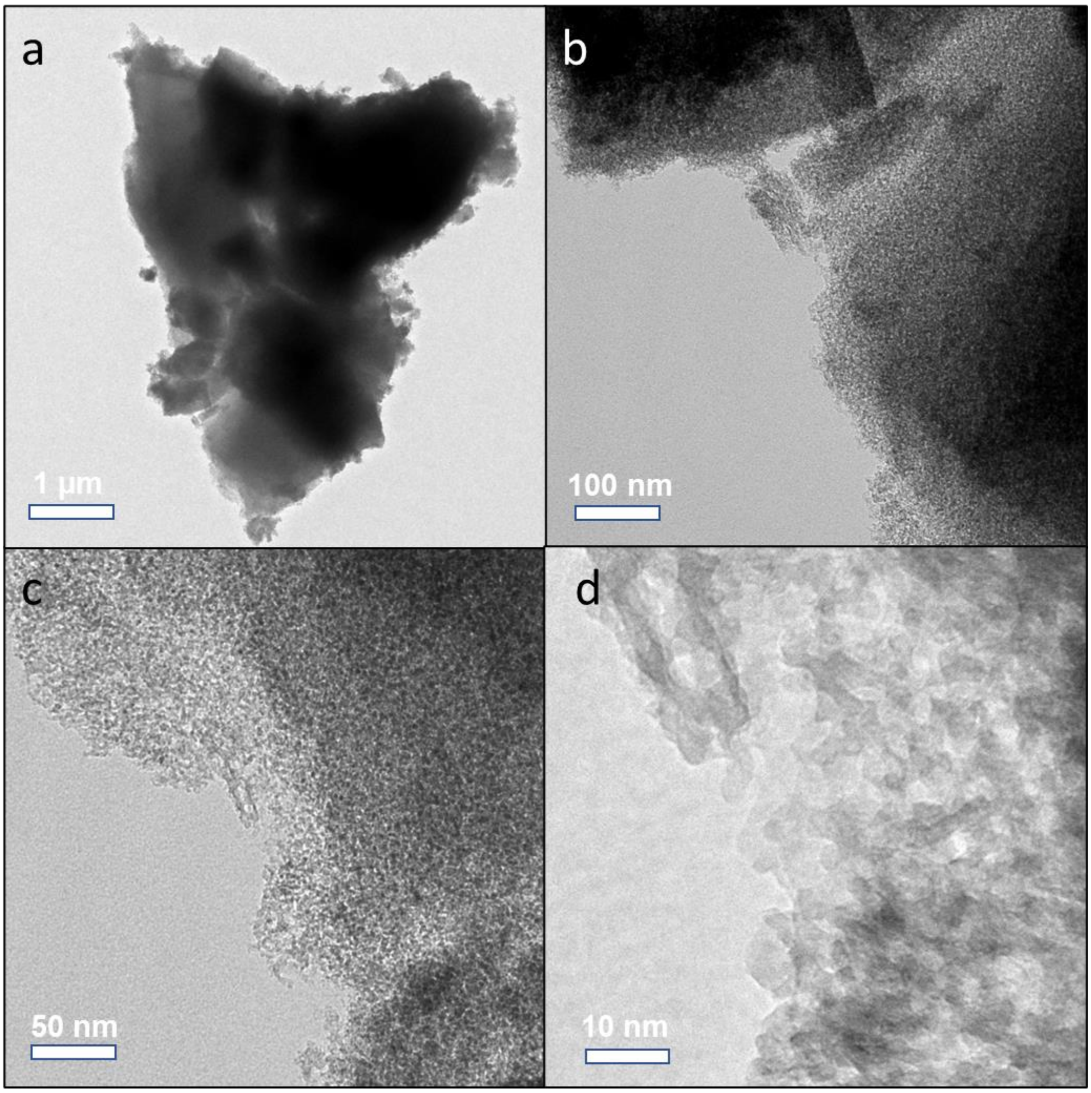

Supplementary Figure 3. TEM images of Al_2O_{3-x} at different scales.

Supplementary Note 6: The morphology of Al_2O_{3-x} was characterized by electron transmission microscopy. As shown in the Supplementary Figure 3a, Al_2O_{3-x} is amorphous micron-sized particles. Increasing the magnification reveals the presence of a large number of mesoporous channels in Al_2O_{3-x} (Supplementary Figure 3b and 3c). With further magnification, it can be found that the micro-sized Al_2O_{3-x} are made up of cross-linked 5–10 nm particles stacked together (Supplementary Figure 3d), and the pore diameter between the particles, too, is about 5–10 nm.

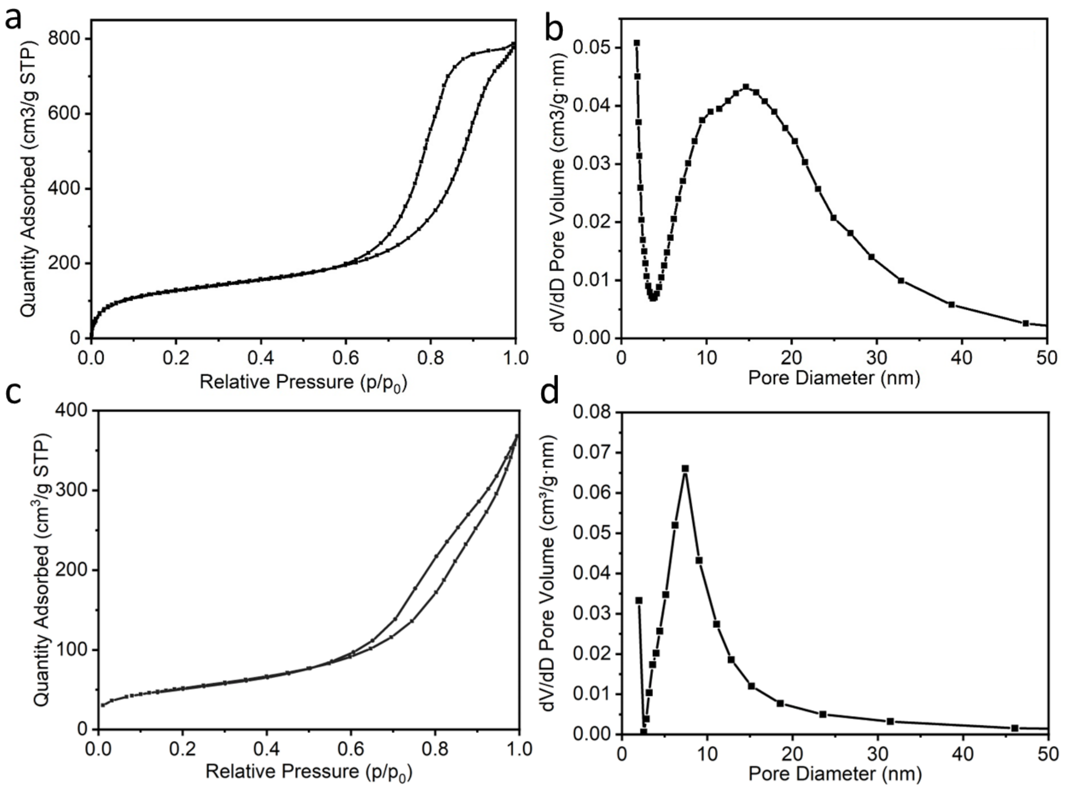

Supplementary Figure 4. N_2 adsorption–desorption isotherms of Al_2O_{3-x} (**a**), and Al_2O_3 (c), and BJH pore size distribution of Al_2O_{3-x} (b) and Al_2O_3 (d).

Supplementary Note 7: The Ar adsorption–desorption isotherm curves of Al_2O_{3-x} shown in Supplementary Figure 4 display typical type IV isotherms with H1 hysteresis loops, which are evidenced by the presence of the capillary condensation step at P/P_0=0.7−1.0 and indicating the existence of mesoporous. The remarkable Ar adsorption at the relative pressure (P/P_0) of 0−0.1 reveals the presence of abundant microporous, as a result of the amorphous structure.[19] Two prominent pore structures are present in the relevant pore size distribution curves, assigned to the mesopores centered at 15 nm, and micropores smaller than 2 nm respectively. Corresponding to the SEM and TEM images, the former should be attributed to the interparticle stacking pores, while the latter intraparticle structural pores.

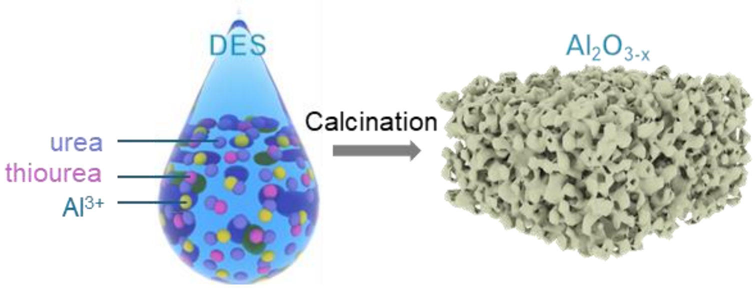

Supplementary Figure 5. The synthesis of Al_2O_{3-x} support.

Supplementary Note 8: Supplementary Figure 5 depicted the as-developed facile method for the synthesis of defective Al_2O_{3-x} support, with a micro-mesoporous structure and abundant Al^{v} species, employing an inexpensive urea-thiourea DES as a solvent.

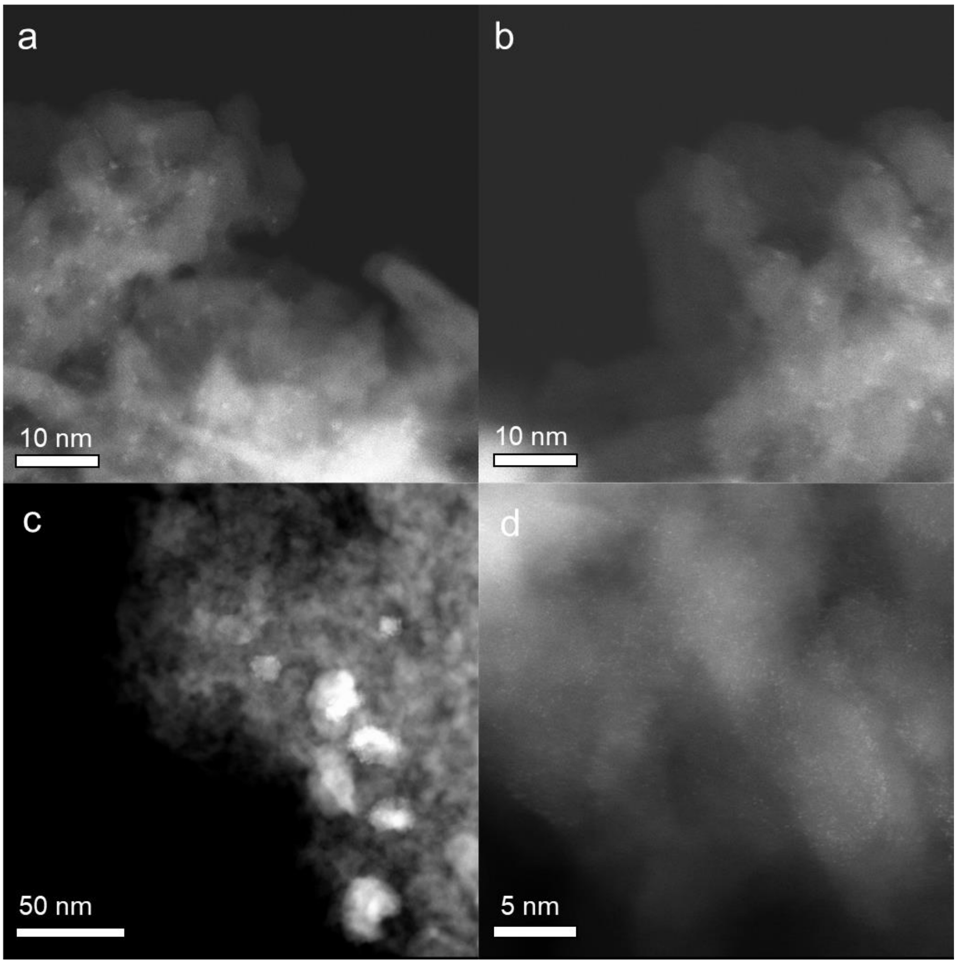

Supplementary Figure 6. HAADF-STEM images of Pd/Al_2O_3-fresh (a, b) and Pd/Al_2O_3-aged (c, d). The Pd/Al_2O_3-aged represents the Pd/Al_2O_3 catalyst after steady-state reaction at 500 °C for 1.5 h. Reaction condition: CH_4:O_2:He= 2:8:90, 50 mg catalyst, GHSV= 60,000 h^{-1}.

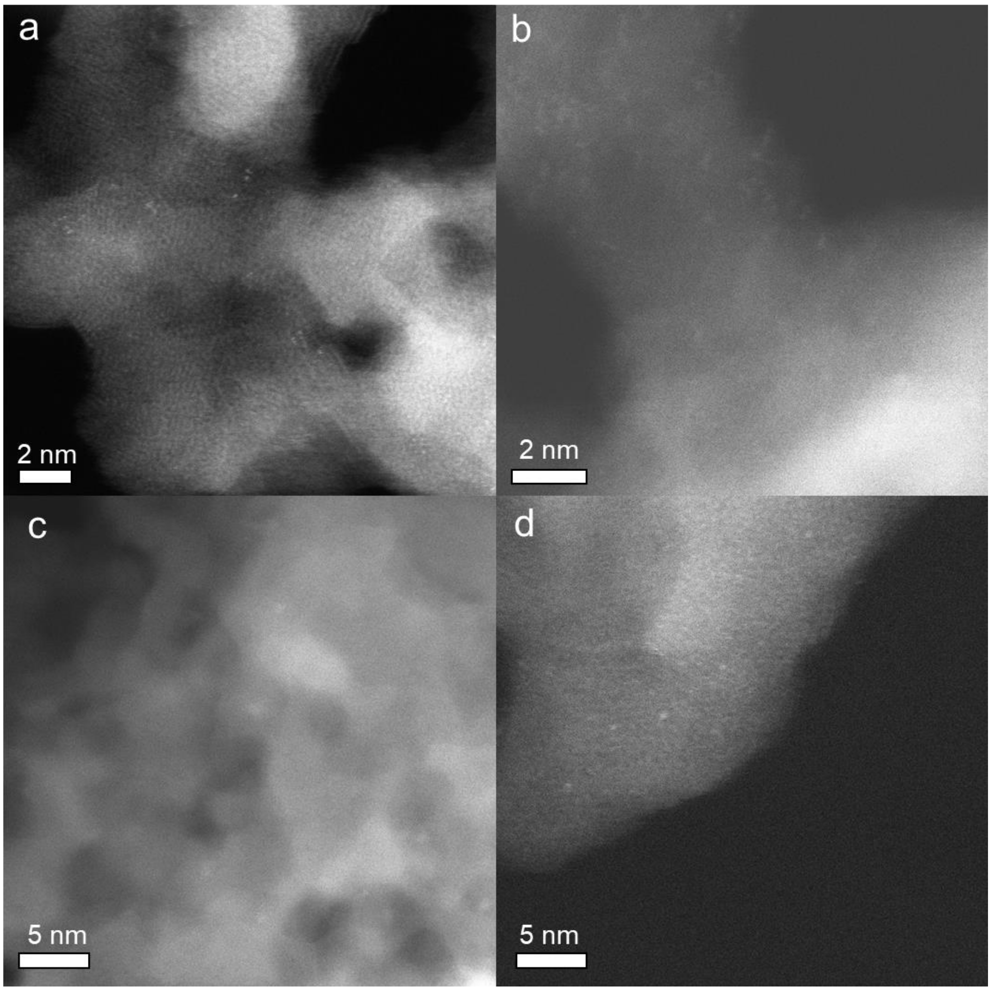

Supplementary Figure 7. HAADF-STEM images of Pd/Al_2O_{3-x}-fresh (a, b) and Pd/Al_2O_{3-x}-aged (c, d). The Pd/Al_2O_3-aged represents the Pd/Al_2O_{3-x} catalyst after steady-state reaction at 500 °C for 1.5 h. Reaction condition: CH_4:O_2:He= 2:8:90, 50 mg catalyst, GHSV= 60,000 h^{-1}.

Supplementary Note 9: In addition to the HAADF-STEM images provided in the main text, more STEM images of Pd at different regions of the catalysts are also presented in Supplementary Figure 6 and Supplementary Figure 7. As demonstrated in Figures 6a and 6b, the PdO on Pd/Al_2O_3 was in the form of small, uniformly dispersed 1–2 nm NPs prior to the reaction. However, as shown in Figure 6c, after aging under methane oxidation conditions for 1.5 h, a large number of Pd species were present in the form of large NPs. This is consistent with the morphology of the Pd active centers on Pd/Al_2O_3 catalyst reported in most of the literature. However, the use of atom-resolution TEM allowed us to see many atomically dispersed Pd species as well on Al_2O_3. Such highly dispersed Pd species were not present in the fresh catalyst and were generated during the aging process. As discussed in the main text, the generation of these single-atom Pd may be associated with the mobility of the Pd species as well as the exposure of Al^{v} on Al_2O_3 during reaction.

Certainly worth noting is the potential for the electron beam itself to induce reduction and aggregation

of Pd species. During the HAADF-STEM testing process, prolonged exposure indeed resulted in an increase in the size of Pd NPs. Therefore, during our operations, we mitigated the impact of the electron beam on the catalyst by reducing the measurement time in the test region.

Because of the poor electrical conductivity and hierarchical porous structure of Al_2O_{3-x}, as well as the limited depth of focus of atom-resolution TEM, Pd single atoms on Pd/Al_2O_{3-x} were rather difficult to image, especially considering that a fraction of the Pd is present in the micropores. Nevertheless, Pd single atoms can still be observed on Pd/Al_2O_{3-x}-fresh in various regions (Supplementary Figure 7a and 7b), using an appropriate focus. The absence of PdO NPs, which should be more visible than Pd single atoms, demonstrates the high Pd dispersion. However, on the aged Pd/Al_2O_{3-x} catalyst, as shown in the Supplementary Figure 7c, some bright spots of about 1 nm in size appeared in some regions of the catalyst, and they were attributed to PdO_x clusters formed by sintering. In addition, isolated Pd cations can still be found on the support (Fig. 7d), evidencing the coexist of Pd single atoms and clusters after aging.

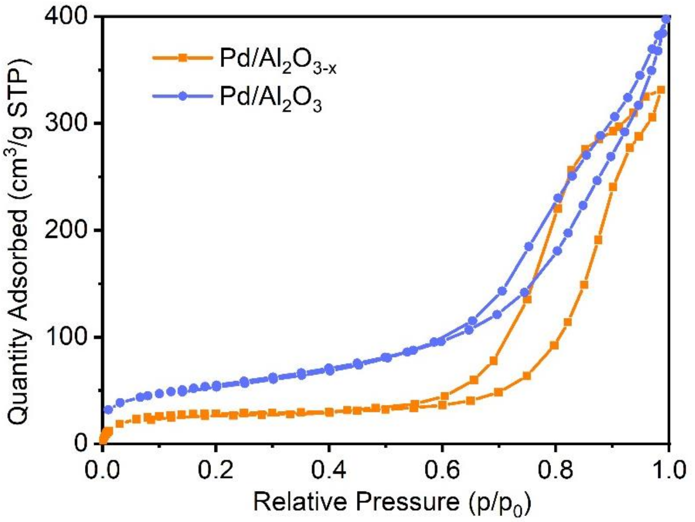

Supplementary Figure 8. N_2 adsorption–desorption isotherms of Pd/Al_2O_{3-x} and Pd/Al_2O_3.

Supplementary Note 10: As shown in Supplementary Figure 8 and Table 3, the loading of Pd on Al_2O_{3-x} resulted in the reduction of specific surface area of the support. This may be due to the strong interaction between Pd and Al_2O_{3-x}, or due to the dissociation of water, absorbed by the impregnation solution, on Al^V sites, that lead to the localized restructuring of the amorphous Al_2O_{3-x} support. However, in general, after loading Pd, Pd/Al_2O_3 and Pd/Al_2O_{3-x} catalysts possess comparable specific surface areas.

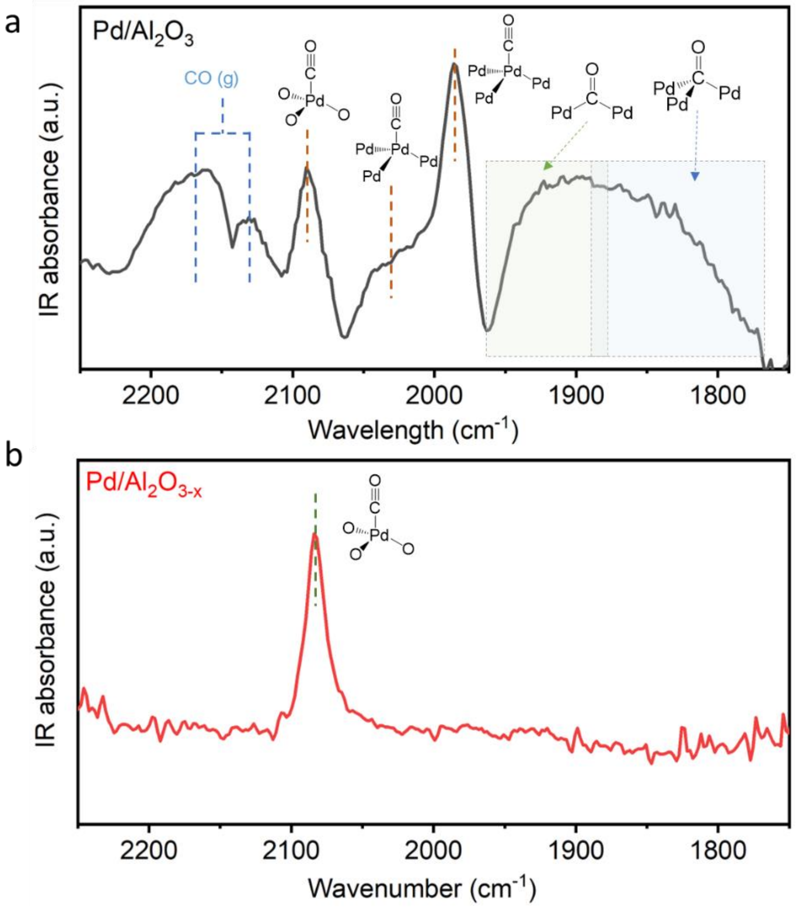

Supplementary Figure 9. FT-IR spectra of CO probe adsorbed on the reduced Pd/Al_2O_3-aged (a) and Pd/Al_2O_{3-x}-aged (b) catalysts.

Supplementary Note 11: Sample-averaged information on the states of the Pd species on the aged catalysts was obtained by FT-IR, using CO as a probe molecule (CO-IR). As clarified in the interpretation of the operando FT-IR results in the main text, CO can work as a probe for surface Pd atoms, whose stretching vibrational frequency is correlated with CO adsorption mode as well as the electronic structure of Pd. Although adsorbed CO is also present in the operando spectra, the preferential adsorption of coexisting other molecules, such as water, formate, carbonic acid, etc., at some specific Pd sites can cause some of the CO to lose some of its functionality as a probe. We therefore used dried CO/He as a probe in combination with IR spectroscopy to characterize the Pd species after aging.

As we have discussed in the main text, Pd stayed in the oxide state during methane oxidation, in which case only linearly adsorbed CO were shown on PdO species. Hence, the catalysts were reduced with hydrogen in situ within the IR cell prior to the measurement. Afterwards, the IR cell was cooled down to 90 K to enhance the adsorption strength of CO at low pressures. The CO-IR spectra of Pd/Al_2O_3 and Pd/Al_2O_{3-x} are shown in Supplementary Figure 9a and Supplementary Figure 9b, respectively. The split double bands, centred at the wavenumber of 2150 and 2174 cm^{-1}, can be attributed to the rovibrational

signals of gaseous CO molecules that are not adsorbed on Pd. The stretching bands of the adsorbed CO are resolved into 2 regions, located at 1950–2100 cm^{-1} and 1800–1950 cm^{-1} respectively. The former region is assigned to the linearly adsorbed CO molecules while the latter correspond to the adsorbed bridged and multi-coordinated CO on Pd sites.[20] CO vibration bands in the linear region can be further classified into two set of peaks in the wavenumber ranges of 1950–2050 cm^{-1} (L1) and 2050–2100 cm^{-1} (L2), assigned to the linearly bonded CO molecules adsorbed over the low coordination sites of metallic Pd NPs, and isolated Pd ions on the surface of alumina, respectively. Additionally, those non-linear regions in the wavenumber range of 1800–1950 cm^{-1} are derived from the superposition of three peaks for the multiply-bound CO molecules over metallic Pd NPs: compressed-bridged, isolated-bridged and tri-coordinated modes. Consistent with the results of online IR spectroscopy, multi-coordinated CO is only observed on Pd/Al_2O_3, indicating the absence of Pd NPs on Pd/Al_2O_{3-x}. The Pd clusters, although also observed on Pd/Al_2O_{3-x}, are at sub-nanometre sizes, making the coordination number of exposed Pd atoms relatively lower, which as a result allows it more favourable for the CO molecules to be adsorbed on the surface in the form of linear bonding.[20] In addition, the CO molecule adsorbed on the single-atom Pd over Pd/Al_2O_3 exhibits a slightly higher vibrational frequency than that on Pd/Al_2O_{3-x}. One possible reason is that the anchored Pd atoms on the crystalline γ-Al_2O_3 may have a relatively higher Pd-O coordination number, leaving Pd in a higher oxidation state.[21]

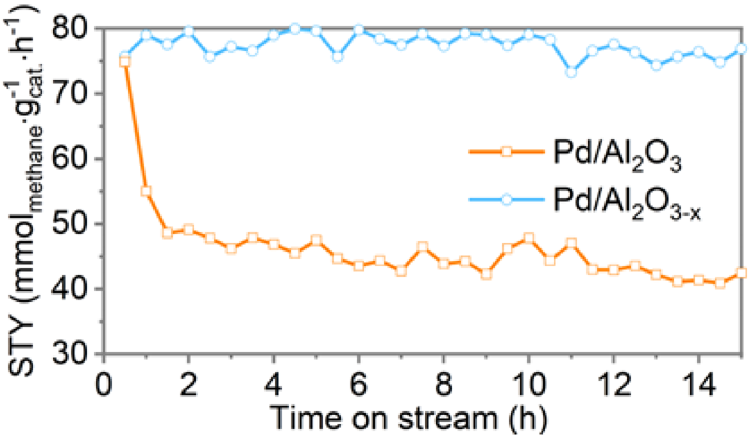

Supplementary Figure 10. Catalytic methane oxidation activities of Pd/Al_2O_3 and Pd/Al_2O_{3-x} plotted against time on stream at 500 °C. CH_4 : O_2 : He = 2:8:90, reaction temperature: 500 °C, and GHSV = 120,000 mL·g_{cat}^{-1}·h^{-1}.

Supplementary Note 12: To study the stability of the catalysts under the operating conditions of natural gas vehicle (NGV) engines, long-term catalytic methane oxidation reaction tests were performed on Pd/Al_2O_3 and Pd/Al_2O_{3-x} at 500 °C. Considering that partial deactivation of the catalyst may not necessarily observed at this reaction temperature when the methane conversion stay 100 %, we further increased the gas hourly space velocity of the catalytic reaction to 120,000 h^{-1} to ensure a lower methane conversion. In this case, the catalytic performance of Pd/Al_2O_3 and Pd/Al_2O_{3-x} are shown in Supplementary Figure 10. It can be observed that Pd/Al_2O_{3-x} exhibits a certain degree of activation in the initial stage of the reaction, followed by relative stability, while Pd/Al_2O_3 experiences rapid deactivation from the beginning of the reaction and reaches a steady state after 2 h.

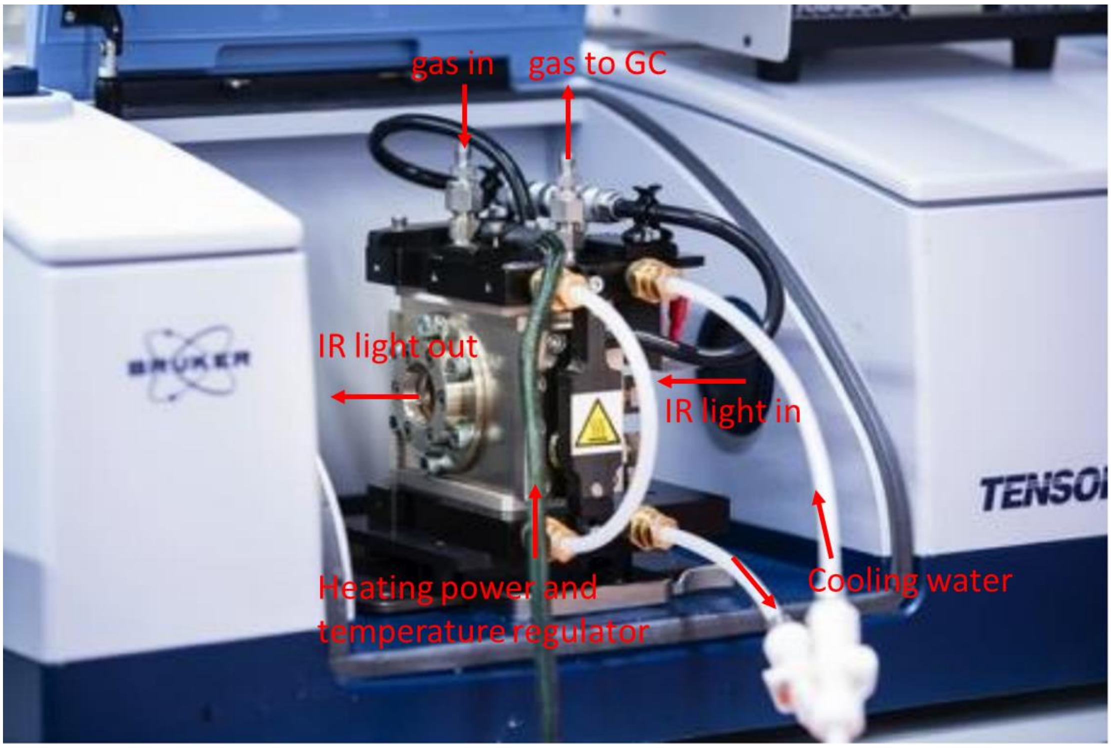

Supplementary Figure 11. Photograph of the Specac High Temperature High Pressure operando transmission infrared spectroscopy cell in the Bruker Tensor 37 FT-IR spectrometer.

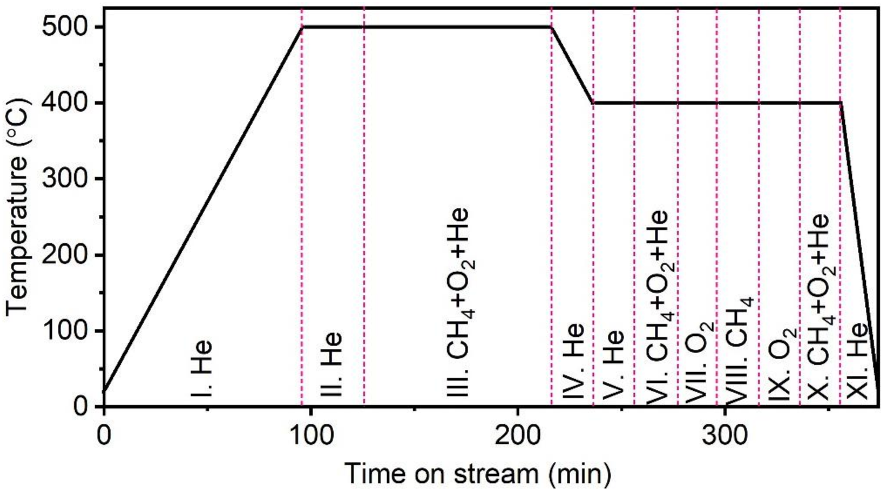

Supplementary Figure 12. The catalytic reaction program of the operando FT-IR spectroscopy experiments.

Supplementary Note 13: The methane oxidation experiments were performed in a Specac High Temperature transmission IR reaction cell (as depicted in Supplementary Figure 11). Prior to the measurement, the catalyst powders were pressed into wafers of approximately 16 mm in diameter, around 0.1 mm in thickness, weighing about 25 mg. These self-supported catalyst wafers were made with a Specac Laboratory Pellet Press, a diaphragm vacuum pump and around 2.5 t of pressure. To gain insight into the catalytic methane oxidation reaction mechanisms over PdO_x NPs, clusters and single atoms, a series of operando IR experiments were designed. The catalytic reaction program of the operando IR experiment is shown in Supplementary Figure 12 and Supplementary Table 5, which contains respectively the pretreatment of catalyst in He, at 500 °C (I and II); aging in the reaction atmosphere and at 500 °C (III); cooling to 400 °C in He (IV and V); a successive gas switch to the reaction mixture (VI), oxygen (VII), methane (VIII), oxygen (IX) and reaction mixture (X), respectively, at 400 °C; and finally cooling to room temperature in He (XI). Meanwhile, IR spectra in the wavenumber range of 4000-1000 cm^{-1} and a resolution of 2 cm^{-1} were acquired every minute, using a DTGS detector. The reactants were introduced through Bronkhorst EL-FLOW Mass Flow Controllers. The flow rate of the reactants during each process is shown in Supplementary Table 3. To simulate real catalytic reaction conditions, the gas hourly space velocity was controlled to be 60,000 $mL \cdot g_{cat}^{-1} \cdot h^{-1}$. He was used as atmosphere for the background of all spectra. To make the IR signal of the surface species more visible and to eliminate the contribution of the support itself to the IR spectra, from the spectra obtained in process VI-X we subtracted the spectrum collected in process V, i.e., the spectrum of the catalysts in He, at 400 °C (Process V).

In the designed reaction procedure, the catalysts were first dehydrated under He to clean the catalyst surface and to prevent surface impurities from interfering with the IR spectrum. The catalysts were then

aged at 500 °C for 90 min to allow the Pd active centre to evolve into particles or clusters. After that, the aged catalyst was cooled to 400 °C in He to obtain the IR spectra of the catalysts themselves at this temperature, which were subtracted as background in the IR spectra collected later. Next, the catalysts were successively exposed to the reaction mixture or individual reactants to examine the time-resolved response of intermediates on different catalyst active centres to different feedstocks.

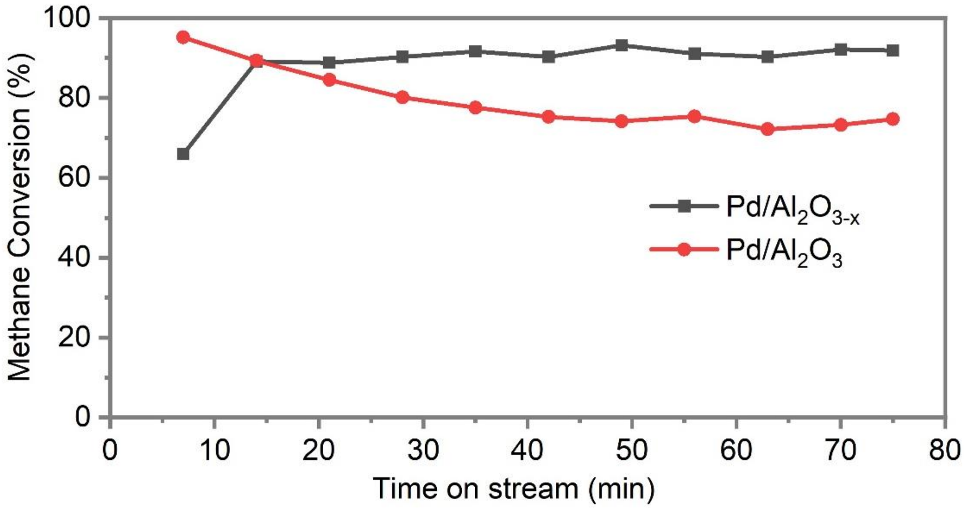

Supplementary Figure 13. The catalytic methane oxidation activities of Pd/Al_2O_3 and Pd/Al_2O_{3-x} plotted against time at 500 °C, collected by the online GC system coupled to the FT-IR spectrometer. CH_4 : O_2 : He = 2:8:90, reaction temperature: 500 °C, and GHSV = 60,000 mL·g_{cat}^{-1}·h^{-1}.

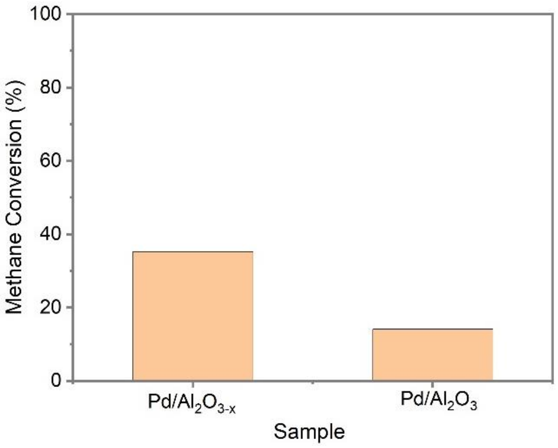

Supplementary Figure 14. The catalytic methane oxidation activities over Pd/Al_2O_3 and Pd/Al_2O_{3-x} at 400 °C, collected by the online GC system coupled to the FT-IR spectrometer. $CH_4 : O_2 : He$ = 2:8:90, reaction temperature: 400 °C, and GHSV = 60,000 $mL{\cdot}g_{cat}^{-1}{\cdot}h^{-1}$.

Supplementary Note 14: On-line product analysis was performed with an Interscience custom-built Global Analyzer Solutions (G.A.S) Compact $GC_{4.0}$ gas chromatograph (GC) with an injection time-resolution of 3.5 min. Methane and CO_2 were detected and quantitatively analyzed using the Flame Ionization Detector (FID) and Thermal Conductivity Detector (TCD) respectively. At a set CH_4/O_2 ratio, the complete oxidation products CO_2 and water are the only products. Therefore, the activity of the catalyst was evaluated by methane conversion:

Conversion (CH_4) = $(C_0-C_t)/C_0*100\%$

where C_0 and C_t represent the initial and post-catalytic reaction methane concentrations, respectively. The variation of catalytic activity with time during the evolution of Pd species at 500 °C is shown in Supplementary Figure 13. The activity of Pd/Al_2O_3 and Pd/Al_2O_{3-x} is worse compared to that obtained in the fixed-bed reactor because in the operando IR spectrometer, the catalyst is loaded in the form of wafer in the IR reaction cell and the catalytic reaction rate is limited by mass transfer. However, a deactivation of Pd/Al_2O_3 was still revealed from Supplementary Figure 13, while the catalytic stability and activity of Pd/Al_2O_{3-x} are both higher in comparison. The Pd/Al_2O_3 and Pd/Al_2O_{3-x} catalysts reached 35.2 and 14.7 % methane conversion after stabilization at 400 °C and in the reaction atmosphere, respectively (shown in Supplementary Figure 14).

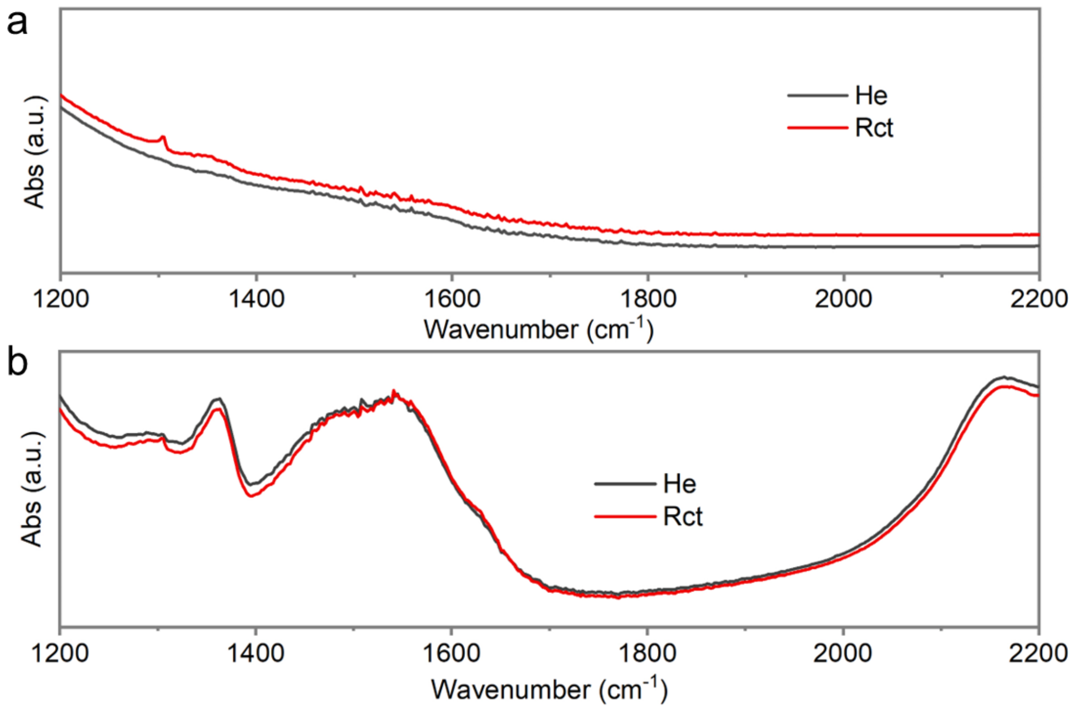

Supplementary Figure 15. Operando FT-IR spectra of Pd/Al_2O_3 (top) and Pd/Al_2O_{3-x} (bottom) in the He and reaction atmosphere (Rct) under steady states. The reaction atmosphere is $CH_4 : O_2 : He = 2:8:90$, reaction temperature = 400 °C, and GHSV = 60,000 $mL \cdot g_{cat}^{-1} \cdot h^{-1}$.

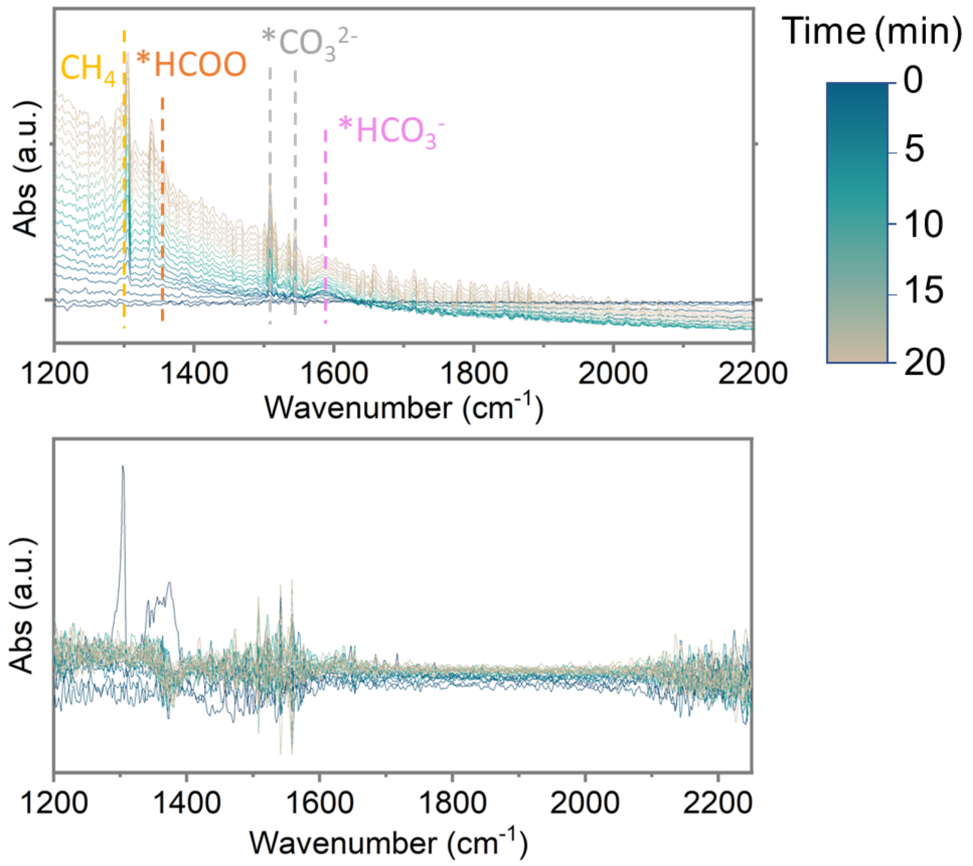

Supplementary Figure 16. Time-resolved operando FT-IR spectra of Pd/Al_2O_3 (top) and Pd/Al_2O_{3-x} (bottom) in the O_2-to-reaction atmosphere switching processes. The IR spectra of Pd/Al_2O_3 and Pd/Al_2O_{3-x} in the atmosphere of He was subtracted. The reaction atmosphere is CH_4 : O_2 : He = 2:8:90, reaction temperature = 400 °C, and GHSV = 60,000 mL·g_{cat}^{-1}·h^{-1}.

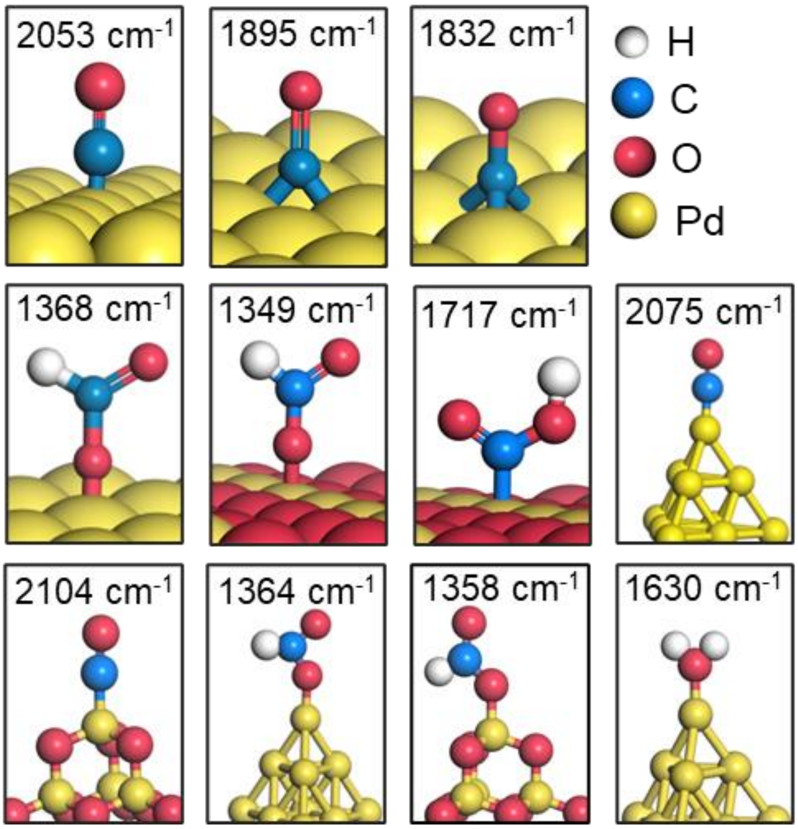

Supplementary Figure 17. Some plausible structures of the methane oxidation intermediates adsorbed on Pd and PdO NPs or clusters, inferred from the IR absorption bands.

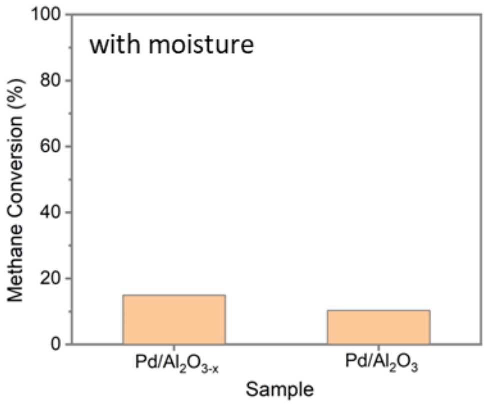

Supplementary Figure 18. The catalytic methane oxidation activities in the presence of moisture over Pd/Al_2O_3 and Pd/Al_2O_{3-x} at 400 °C, collected by the online GC system coupled to the FT-IR spectrometer. CH_4 : O_2 : He : H_2O = 2:8:86:4, reaction temperature: 400 °C, and GHSV = 60,000 mL·g_{cat}^{-1}·h^{-1}.

Supplementary Note 15: In section 2.3 of the main text, we observed the accumulation of adsorbed water molecules on Pd/Al_2O_{3-x} during in situ FT-IR experiments in CH_4/O_2 switching. This may imply that water molecules are difficult to desorb from atomically dispersed Pd species, leading to a poisoning effect. Considering this, we introduced 4% water vapor during the operando FT-IR tests. After reaching steady state, the catalytic methane oxidation activities of both catalysts, recorded by online gas chromatography, are displayed in Supplementary Figure 18 Compared to the conditions without water vapor, the catalytic methane conversion rate of Pd/Al_2O_{3-x} decreased from 35.2% to 15.0%, while that of Pd/Al_2O_3 decreased from 14.2% to 10.3%. This indicates that atomically dispersed Pd species are still susceptible to poisoning by water vapor.

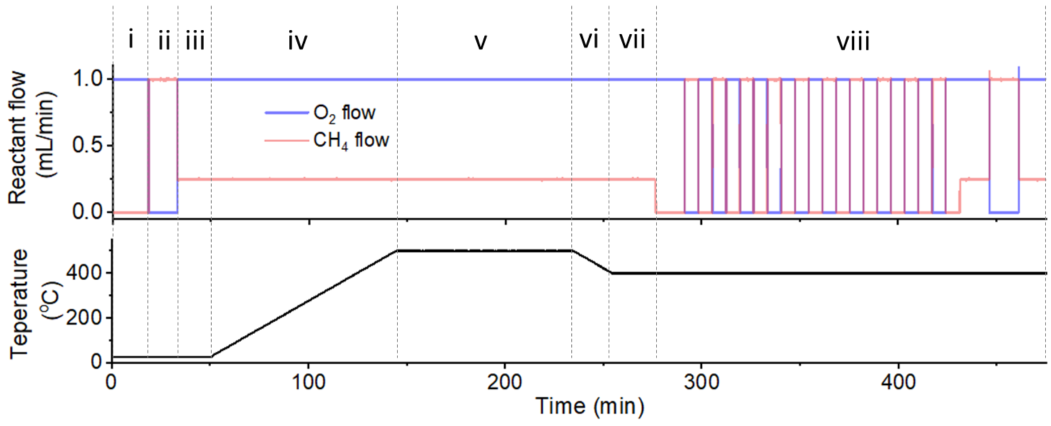

Supplementary Figure 19. The catalytic reaction program for the operando QEXAFS experiments.

Supplementary Note 16: Operando X-ray spectroscopy experiments were performed in a home-built cell coupled with a gas and temperature control system. Glass capillaries with an inner diameter of 1 mm were used as reactors. Catalysts of a grain size of 36–75 μm were filled into the capillary with a bed thickness of 1 cm. The mass of the filled catalyst was about 10 mg. Quartz wool was filled into both ends of the catalyst bed to prevent the catalyst from moving. Graphite ferrules hold a thermocouple in place in the gas stream, and against the catalyst ensuring a correct temperature measurement. Gas flows were preheated to 80 °C.

An automated 3-way VICI valve system was custom built to enable quick gas switching. 10 % CH_4/He was attached to a single valve inlet, while 10 % CH_4/He and 10 % O_2/He together were connected to the other single valve inlet. Tubing length was kept at minimum around the cell to ensure short gas switching process time.

A typical catalytic reaction procedure for operando QEXAFS experiment is shown in the Supplementary Figure 19, which consists of (a) the XAS measurement of fresh catalysts at room temperature in O_2 (i), CH_4 (ii) and the CH_4/O_2 mixture (iii) respectively; (b) operando XAS measurement during the heating up to 500 °C in a reaction atmosphere (iv); (c) catalyst evolution in the reaction atmosphere at 500 °C (v); (d) cooling down to the reaction temperature of interest, 400 °C (vi); (e) reaction at 400 °C (vii); and (f) modulation excitation in the alternative pulses of CH_4, O_2 or CH_4/O_2 mixture (viii). Pd K-edge XANES and EXAFS recorded for the samples were acquired at a time resolution of one second throughout the catalytic reaction. At the same time, the major products were recorded by mass spectrometry at a time resolution of 2.5 s.

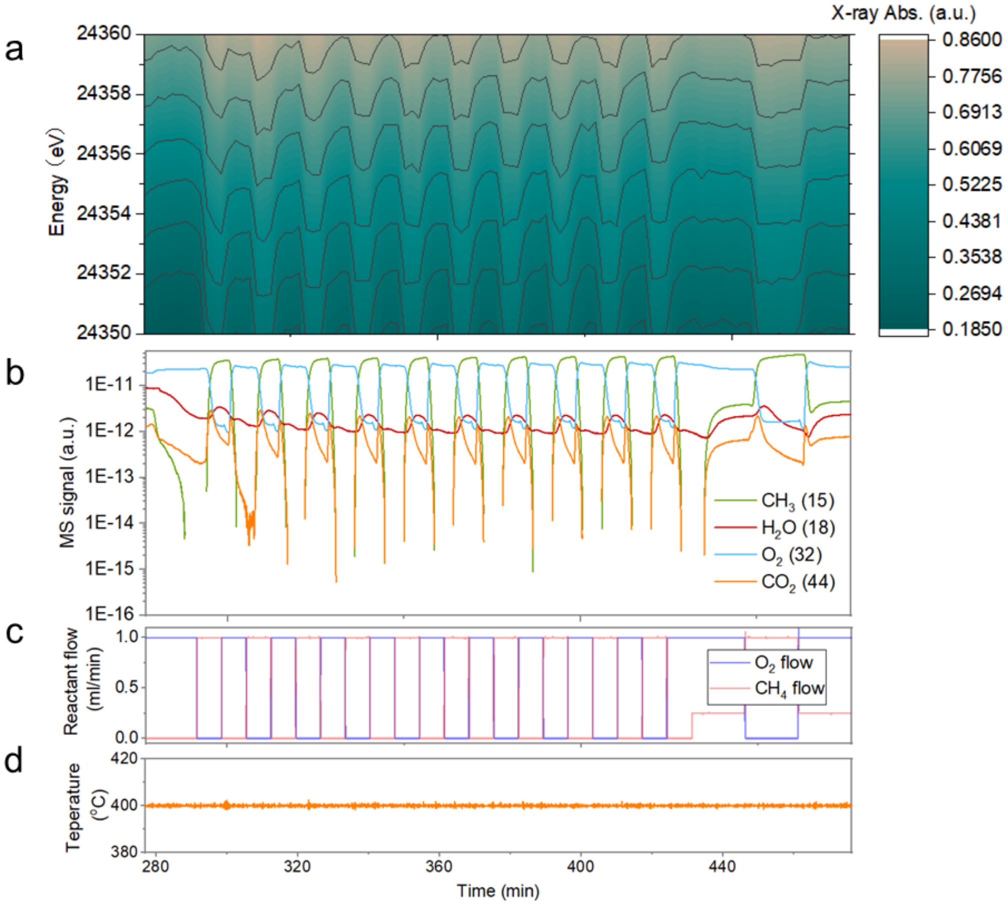

Supplementary Figure 20. (a) The operando Pd K-edge XANES of Pd/Al_2O_3 in the absorption edge region, as a function of time. Corresponding on-line MS results (b), reaction temperature (c) and reactant flows (d) during operando XAS measurements.

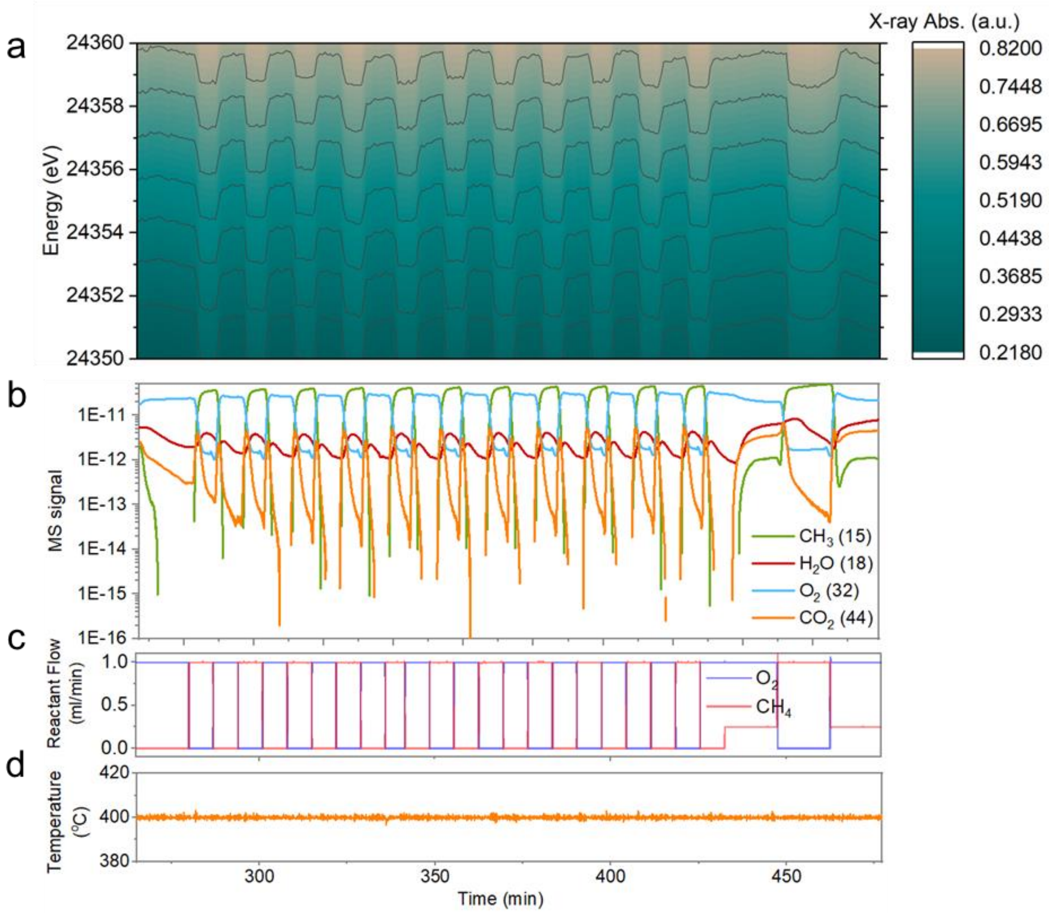

Supplementary Figure 21. The operando Pd K-edge FT-EXAFS (a) and part of the XANES (b) of Pd/Al_2O_{3-x}, as a function of time. Corresponding on-line MS results (c), reaction temperature (d) and reactant flows (e) during operando XAS measurement.

Supplementary Note 17: As has been discussed in the main text, modulation excitation (ME) experiments with 1 s-resolution XAS spectroscopy can assist us in studying the reactivity of individual Pd species.

In addition to the ME experiments with periodic switching between CH_4 and O_2, mixed reactant atmosphere (MIX)-to-O_2, O_2-to-MIX, MIX-to-CH_4 and CH_4-to-MIX switching experiments were also implemented at 400 °C (Supplementary Figure 20c and 21c). Similar to that described in the previous section, the 2-D XANES around the region of the absorption edge (24350-24360 eV) during the ME and afore-mentioned process, are shown in Supplementary Figure 20a and 20a. The more pronounced shift of the X-ray absorption contour over Pd/Al_2O_3 than that over Pd/Al_2O_{3-x} suggests that the Pd species on Al_2O_3 underwent more significant redox transformations or that more Pd species were involved in redox transformations. This has been discussed in detail in the main text.

The oxidation of Pd species on Al_2O_3 was observed during the switch from MIX to O_2 atmosphere. Partially reduced Pd species are present on the PdO NPs active centres under reaction conditions. On Pd

clusters and single atoms (Pd/Al_2O_{3-x}), the oxidation of Pd species is absent after switching to an O_2 atmosphere. After a series of ME cycles, when the reaction atmosphere is switched from O_2 to MIX, Pd/Al_2O_3 is slightly reduced immediately, while Pd/Al_2O_3 is slowly and continuously oxidized. The former is a partial reduction of Pd^{2+} on the Pd NPs once the methane starts to appear; the latter suggests that it is most likely due to the slow sintering of the Pd clusters in the reaction atmosphere.

In the MIX-to-CH_4 atmosphere switch, the Pd species on both Pd/Al_2O_3 and Pd/Al_2O_{3-x} are rapidly reduced to the same oxidation state as in the ME process under methane atmosphere. From the slope of the contour lines in this process it could be concluded that the Pd^{2+}-to-Pd^0 transition is lower on Pd/Al_2O_3 than that Pd/Al_2O_{3-x} , and the latter is even faster than this catalyst itself during the O_2-to-CH_4 switch. There are a number of reasons for this phenomenon, one being that the increase in cluster size after several ME cycles increases its reactivity, and another being that the cluster surface is also in a partially reduced state in the MIX atmosphere, which could be more favourable for the activation of methane. In the CH_4-to-MIX switch, basically the Pd species can be restored to an oxidation state close to that in the O_2 atmosphere. There is however a distinct induction period for the oxidation of PdOx cluster. Or rather, the oxidation of Pd is gradually accelerating on Pd/Al_2O_{3-x}. Considering that CO_2 is being generated rapidly during the process, it can be inferred that the oxidation of partially reduced PdOx clusters by O_2 in the MIX atmosphere is accompanied by a reduction of Pd by CH_4. The latter has a higher reaction rate, making the oxidation of the Pd species slower than Pd/Al_2O_3, apparently. Furthermore, one speculation is that the CH_4 activation on the partially reduced PdOx clusters is favorable and therefore the oxidation of the PdOx clusters is, apparently, slower in the initial stage. Of course, these are only speculations based on the XANES, and more experimental or theoretical efforts are still required to understand the real causes of these changes.

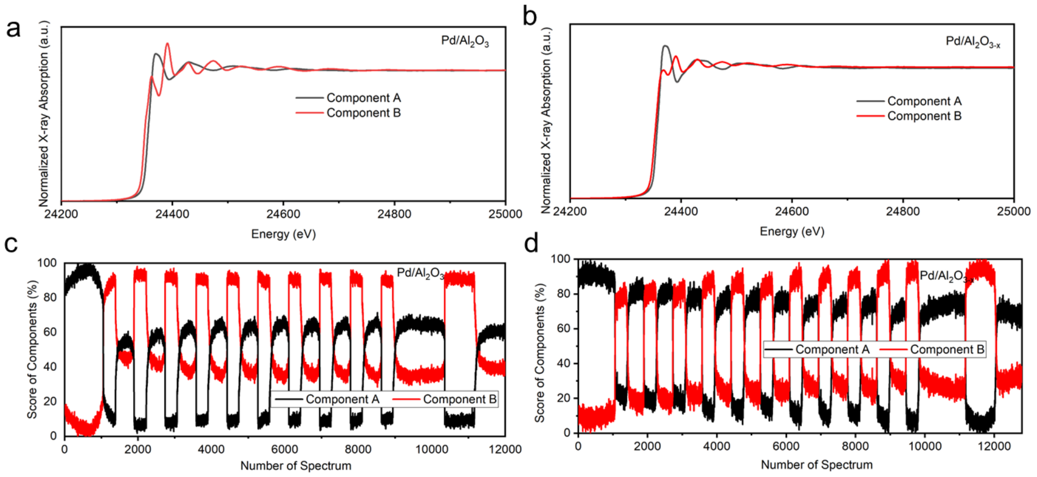

Supplementary Figure 22. EXAFS of the MCR components identified via MCR-ALS analysis of data recorded for Pd/Al_2O_3 (a) and Pd/Al_2O_{3-x} (b). The corresponding variation in contribution (scores) of the components of Pd/Al_2O_3 (c) and Pd/Al_2O_{3-x} (d) as a function of time during methane oxidation reaction at 500 °C.

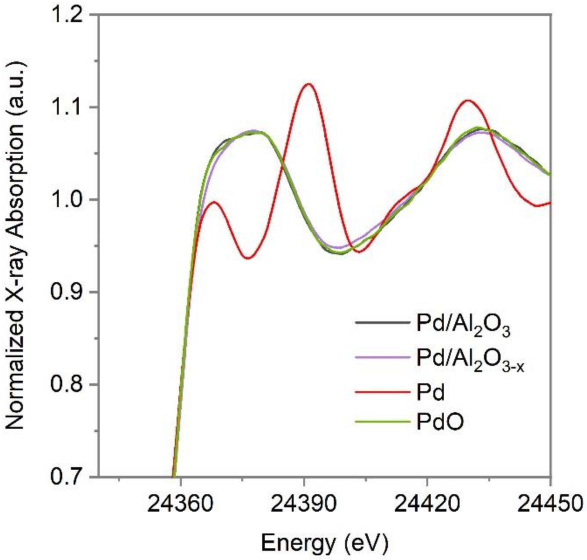

Supplementary Figure 23. XANES of Pd/Al_2O_3, Pd/Al_2O_{3-x}, PdO and Pd. The XANES of PdO and Pd were used as referenced for the LSLCF of the operando QEXAFS data.

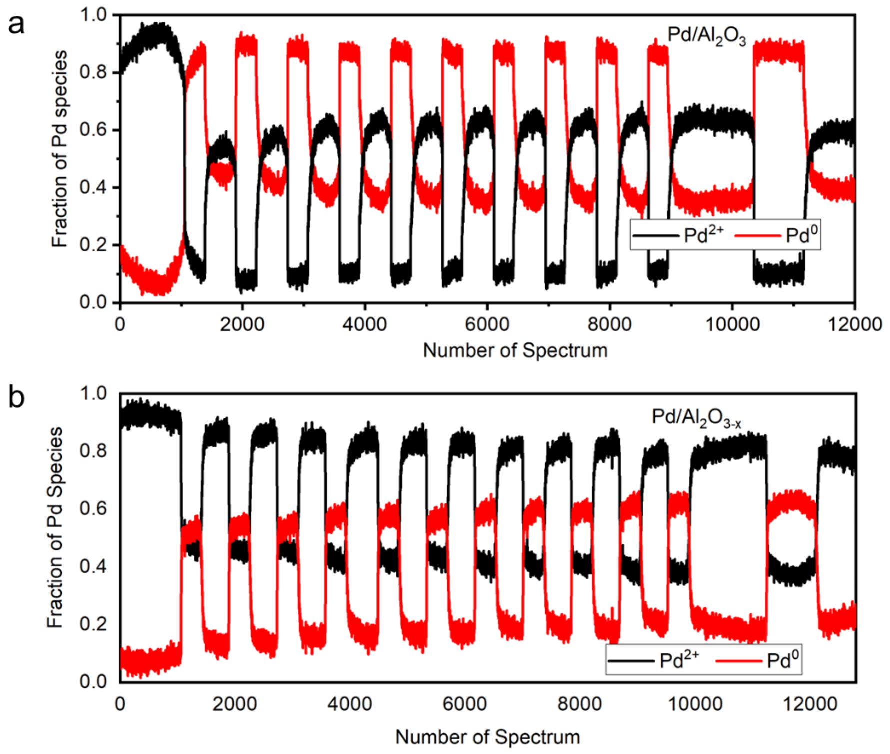

Supplementary Figure 24. Plots of the Pd fraction contents on Pd/Al_2O_3 (a) and Pd/Al_2O_{3-x} (b) during the ME experiments, obtained from linear combination fitting of XANES spectra.

Supplementary Note 18: Multivariate Curve Resolution (MCR) analysis was also employed on the operando X-ray absorption spectra during the ME process. In alternating oxidation-reduction atmospheres, two components were identified for the data of both catalysts, the EXAFS of which are shown in Supplementary Figure 22a and 22b. The variation in the contributions (scores) of each component with time for Pd/Al_2O_3 and Pd/Al_2O_{3-x} are shown in Supplementary Figures 22c and 22d respectively. In order to clarify the changes in the composition of Pd during ME, a least squares linear combination fitting (LSLCF) of reference XANES was performed on the individual components, using PdO and metallic Pd as references (Supplementary Figure 22). The fittings were performed in the energy range of 24340-24390 eV, the weights of components were forced to sum to 1. The obtained fitting results are shown in Supplementary Table 7. Based on the linear combination fitting results and the score plots of the components, we obtained the plots of the fraction of Pd^0 and Pd^{2+} species on Pd/Al_2O_3 and Pd/Al_2O_{3-x} with time, which are shown in Supplementary Figure 24a and 24b, respectively. These results have been partially discussed in the main text. It is still noteworthy, however, that a large number of Pd^{2+} species on Pd/Al_2O_3 are converted to Pd^0 species during the first O_2-to-CH_4 pulse switch, and that the resultant Pd^0 species are no longer changed during the subsequent alternative CH_4/O_2 pulses. This phenomenon occurs only on Pd NPs (Pd/Al_2O_3), implying that the non-oxidizable Pd^0 species is present in the interior of the PdO NPs and that the diffusion of oxygen atoms from the surface to the center is

kinetically slow. In a given gas pulse time, the O atoms could not diffuse completely into the NPs, while the subsurface O will be consumed soon in the next CH_4 pulse. In the O_2-to-MIX and MIX-to-CH_4 switching process, no differences from CH_4/O_2 switching are observed. While in the CH_4-to-MIX switching, a slower Pd^0-to-Pd^{2+} is observed, which has been discussed above. As discussed in section 2.2 of the main text, ME experiments result in irreversible cumulative changes to the active centres with increasing cycle numbers. Upon recovery from ME cycling to reaction conditions and achieving steady state, the activities of both catalysts decreased in varying degrees (as shown in Supplementary Figures 20b and 21b).

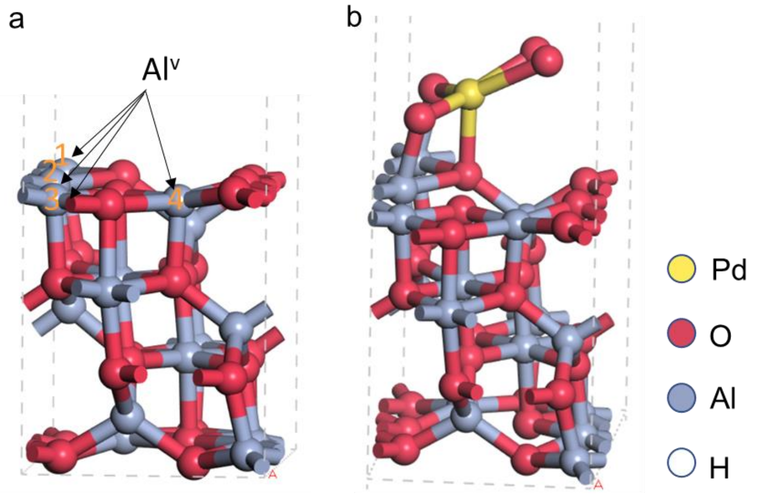

Supplementary Figure 25. Illustration of (a) the (1 0 0) facet of γ-Al_2O_3, Alv species was exposed on the surface. (b) The optimized structure of Pd_1/Al_2O_{3-x}.

Supplementary Note 19: Creating a model of an amorphous alumina surface as the one we used in this study is technically challenging. [22] This is due to several factors: (i) reproducing the structure of an amorphous alumina slab is often difficult; (ii) an amorphous alumina slab obtained through annealing may not possess sufficient surface Al^V, which is believed to be the site required to fix single-atom Pd[23]; and (iii) alterations in the support's geometry may occur when the unstable amorphous alumina structure undergoes surface reactions, which can disrupt the calculation of the necessary adsorption energies.

Considering the results shown in XRD patterns that the synthesized defective aluminum oxide still exhibits low-crystallinity gamma-phase Al_2O_3 when Pd is added, we employed gamma-alumina cell as a model of carrier. Among the various surfaces of gamma-aluminum oxide explored by previous researchers[24-26], the (100) surface is featured with the exposure of a significant number of Al^V sites (Supplementary Figure 25a), which meets our requirement for a slab with sufficient Al^V. Additionally, previous studies on the hydroxyl coverage of different facets of γ-Al_2O_3 revealed that the (100) facet has a hydroxyl coverage of zero at the required reaction temperature (400 °C) [24-26]. Therefore, in our subsequent calculations, the hydroxyl groups on the aluminium oxide surface were disregarded. Previous research has demonstrated that noble metals are linked to Al^v through oxygen bridges and consequently affixed to the Al_2O_3 surface[10, 27]. Four distinct Al^V sites can be found on the Al_2O_3 (1 0 0) facet, which are respectively labelled as 1-4, as shown in Supplementary Figure 25a. The adsorption energies of a PdO_2 molecule on these different Al^V sites were tested to obtain the most stable configuration of single-atom Pd on Al_2O_3. The obtained adsorption energy values are listed in Supplementary Table 8.

The adsorption energy values indicated that the coordination environment at Al^V sites 1 and 2 is most favourable for anchoring single Pd atoms. Therefore, this structure is selected as the initial model for subsequent calculations. Furthermore, as discussed in section 2.3 of the main text, the adsorption of O_2

assists in the desorption of H_2O on single Pd sites. Therefore, the Pd single atom site with an adsorbed O_2 molecules (as shown in Supplementary Figure 25b) has been selected as the initial active centre structure for the catalytic cycles.

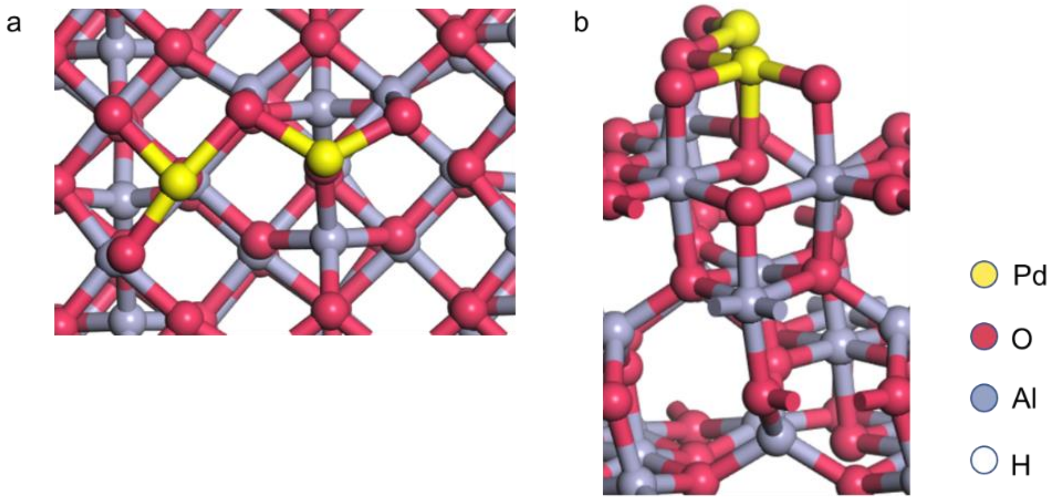

Supplementary Figure 26. Top (a) and side (b) views of the Pd_2O_4 adsorbed on the Al_2O_3 (100) surface.

Supplementary Note 20: To further validate the strong interactions between Al^V sites on Pd centres, a geometric optimization of Pd_2O_4 adsorbed on the Al_2O_3 (1 0 0) slab was performed. As shown in Supplementary Figure 26a and 27b, Pd_2O_4 tends to be anchored on Alv sites through oxygen bridges, simultaneously consuming the exposed Al^V sites. This suggests that in the presence of Al^V sites, multi-core Pd active centres are more inclined to disperse on the Al_2O_3 support rather than self-nucleate into clusters.

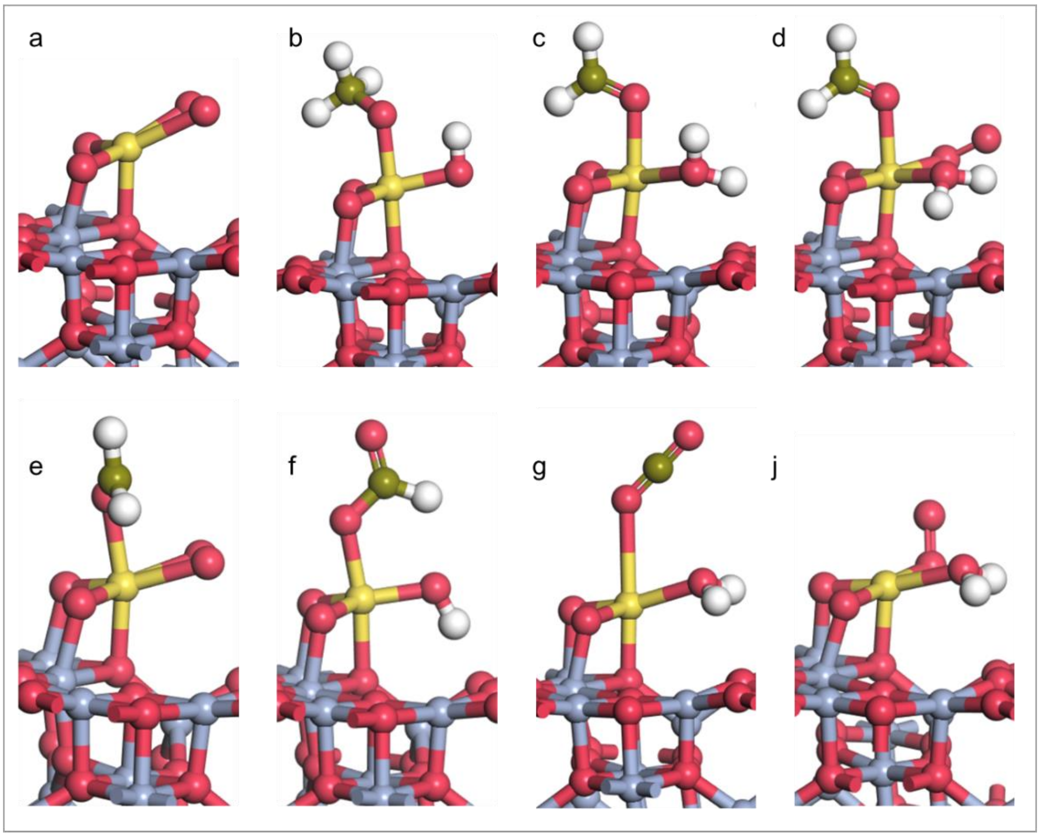

Supplementary Figure 27. Structural illustration of the individual intermediates on the formate branch of the -OCH_3 pathway, obtained through DFT calculations. The gray, red, yellow, white and brown spheres represent Al, O, Pd, H and C atoms, respectively.

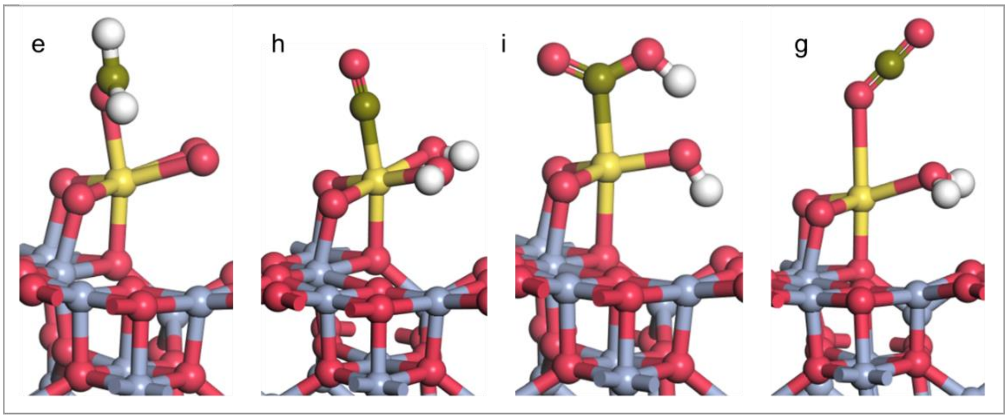

Supplementary Figure 28. Structural illustration of the individual intermediates on the CO branch of the -OCH_3 pathway, obtained through DFT calculations. The gray, red, yellow, white and brown spheres represent Al, O, Pd, H and C atoms, respectively.

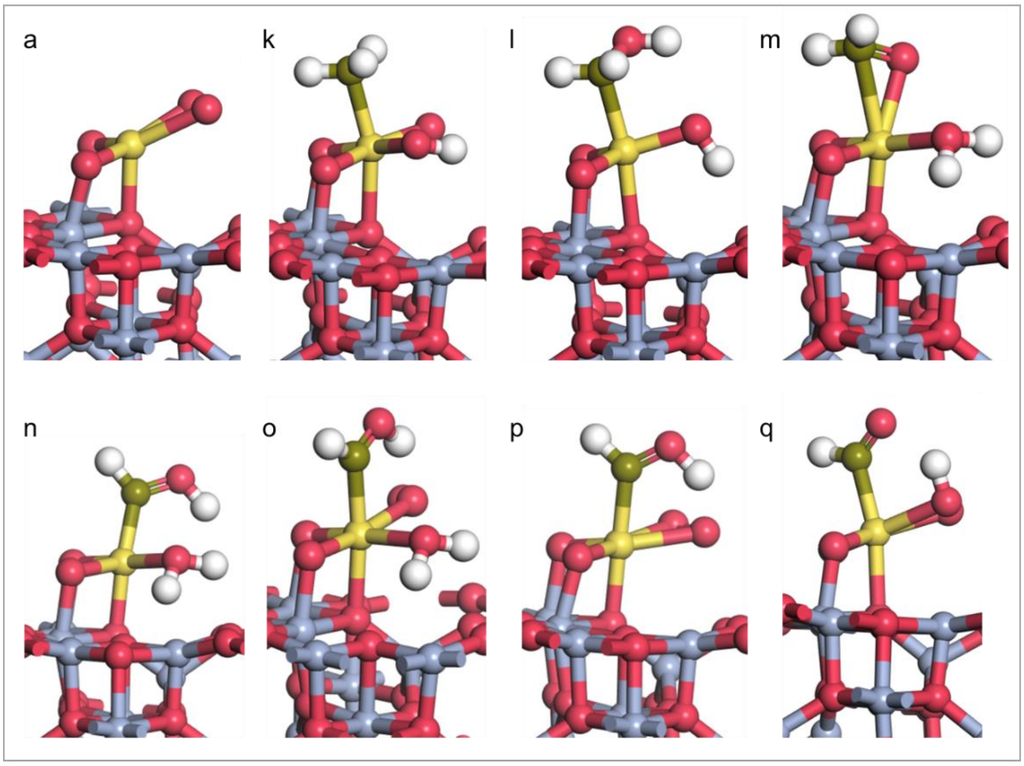

Supplementary Figure 29. Structural illustration of the individual intermediates on the -CH_2OH branch of the -CH_3 pathway, obtained through DFT calculations. The gray, red, yellow, white and brown spheres represent Al, O, Pd, H and C atoms, respectively.

Supplementary Note 21: The complete oxidation of methane to CO_2 involves multiple elementary steps, and previous studies have explored various plausible reaction pathways[28-31]. However, the investigation of reaction pathways specifically on single-atom Pd catalysts remains relatively limited. Expanding upon previous discoveries, we have classified the mechanism of methane oxidation on single-atom Pd into two distinct pathways: the -OCH_3 pathway and the -CH_3 pathway. The former involves the presence of both CO and formate branches, while the latter encompasses the carbide branch and the -CH_2OH branch. Within these pathways, there exists a potential for mutual conversion between intermediates. The reaction network formed by the interconnection of these four pathways, as obtained through DFT calculations, has already been presented and discussed in the main text. Among them, the carbide branch is excluded from the reaction network due to unfavourable thermodynamics, which will be further discussed below. The optimized geometries of the individual intermediates depicted in Figure 5 in the main text are respectively shown in Supplementary 27, 28, and 29. The labels in Supplementary Figure 27, 28 and 29 correspond to that of the intermediates in Figure 5 in the main text as well.

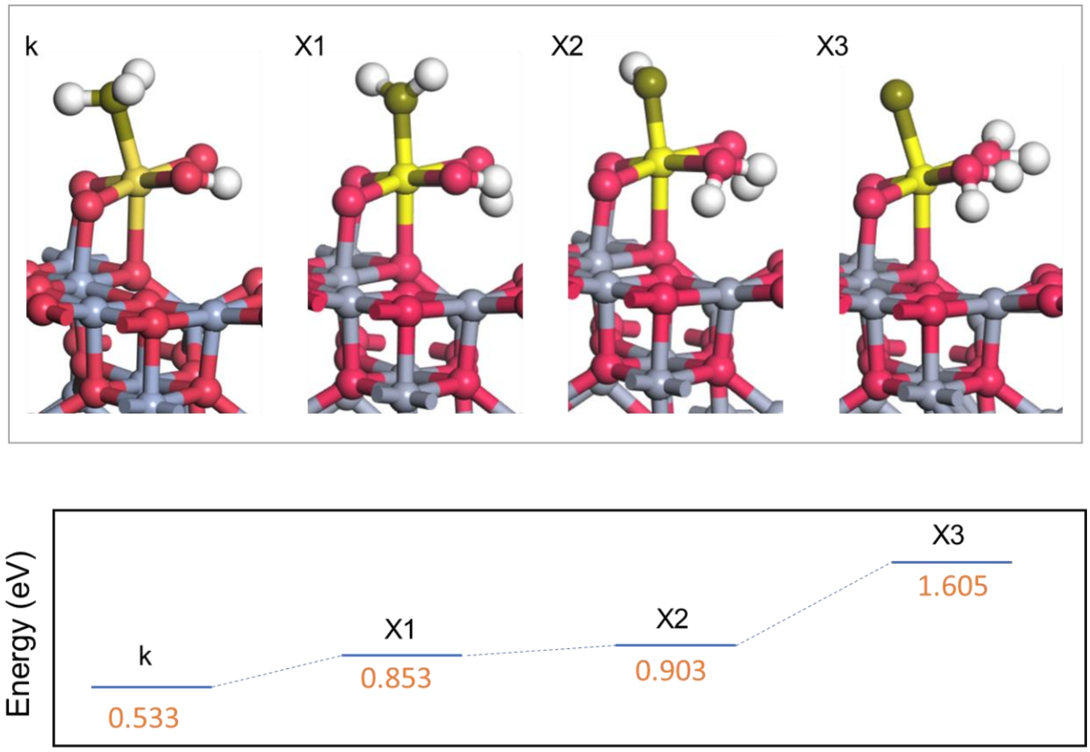

Supplementary Figure 30. Constrained geometric structure optimization of the individual intermediates on the carbide branch of the -CH_3 pathway. Illustration of the obtained structures of the intermediates (top), and the respective relative Gibbs free energy changes (bottom). The gray, red, yellow, white and brown spheres represent Al, O, Pd, H and C atoms, respectively.

Supplementary Note 22: The carbide pathway is commonly acknowledged as a fundamental mechanistic pathway involved in the complete oxidation of methane on PdOx NPs[28, 30-32]. Nevertheless, we observed that the adsorbed *CH_2, *CH, and *C intermediates do not converge into a stable configuration in the structure optimization over Pd single-atom slab. This can likely be attributed to the incapability of fulfilling the coordination requirements of the carbon atom, as *CH_2, *CH, and *C are unable to form a stable bridged or three-fold adsorption structures on the isolated Pd atoms. To further substantiate this perspective, we conducted constrained geometric structure optimization to impose the linear adsorption of *CH_2, *CH, and *C onto single-atom Pd catalysts, with the aim of determining their respective adsorption energies. The resultant geometric configurations and corresponding energies are depicted in Supplementary Figure 30. It is noteworthy that, even under these specified conditions, the energies associated with the pivotal intermediate structures within the carbide pathway exhibit a considerable elevation in comparison to alternative pathways. Consequently, it can be deduced that the carbide pathway is thermodynamically unfavorable on single-atom Pd. This empirical finding effectively aligns with the documented accounts in existing literature pertaining to the heightened capacity of single-atom catalysts to resist coking.

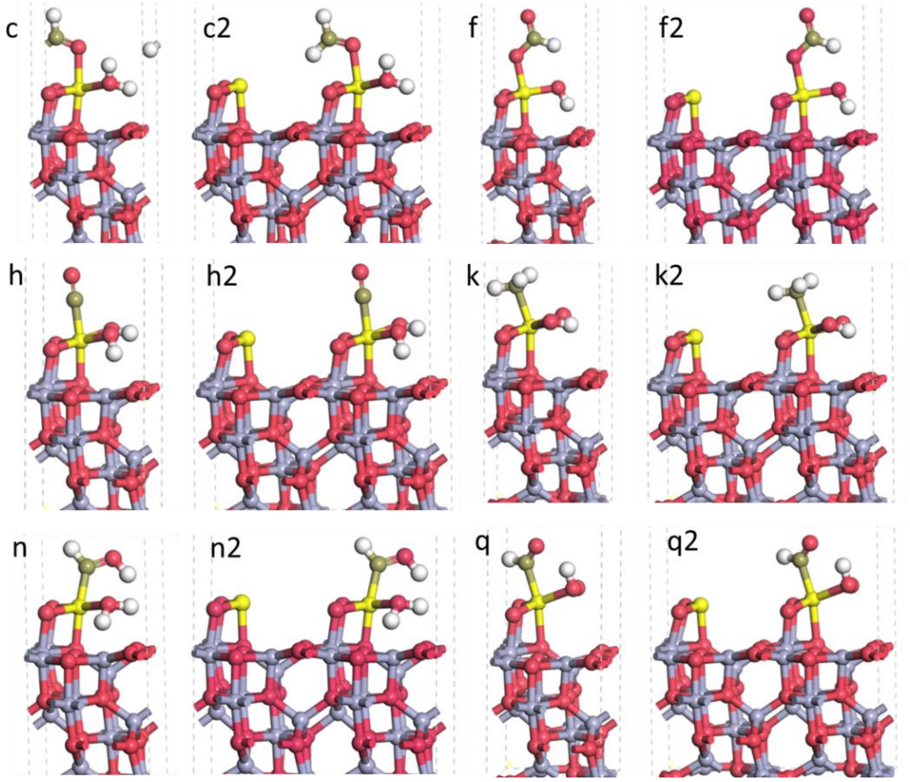

Supplementary Figure 31. Structural illustration of some typical intermediates, adsorbed on single slab (structure c, f, h, k, n and q), and on supercells (structure c2, f2, h2, k2, n2 and q2), obtained through DFT calculations. The grey, red, yellow, white and brown spheres represent Al, O, Pd, H and C atoms, respectively.

Supplementary Note 23: In a slab model with periodic boundaries, there may be interactions between adjacent adsorbed organic intermediates, which could alter the adsorbate geometry and hence the adsorption energy. To investigate whether this effect would be present in our system, especially in the direction A of the slab, where the distance between two adjacent Pd single atoms is only 5.6 Å, we selected several representative intermediates with bulky with large spatial occupancy as study cases, namely intermediates c, f, h, k, n, and q. We performed a replication in the direction A of the initial slab, resulting in a supercell containing two Pd active sites. On one of Pd sites in the supercell, the corresponding functional groups of the intermediates of interest were individually adsorbed. The resulting models were labelled as c2, f2, h2, k2, n2, and q3, respectively. The optimized geometries of these intermediates along with their corresponding single-slab structures are depicted in Supplementary Figure 31, and the obtained Gibbs free energies are listed in Supplementary Table 9. It can be observed that the geometries of the intermediates adsorbed whether on single slabs or on the supercells, are nearly identical. and the corresponding differences in Gibbs free energy remain below 2.8 kJ/mol. Therefore, we can conclude that the energy difference induced by the proximity of adjacent adsorbates is negligible

in our system.

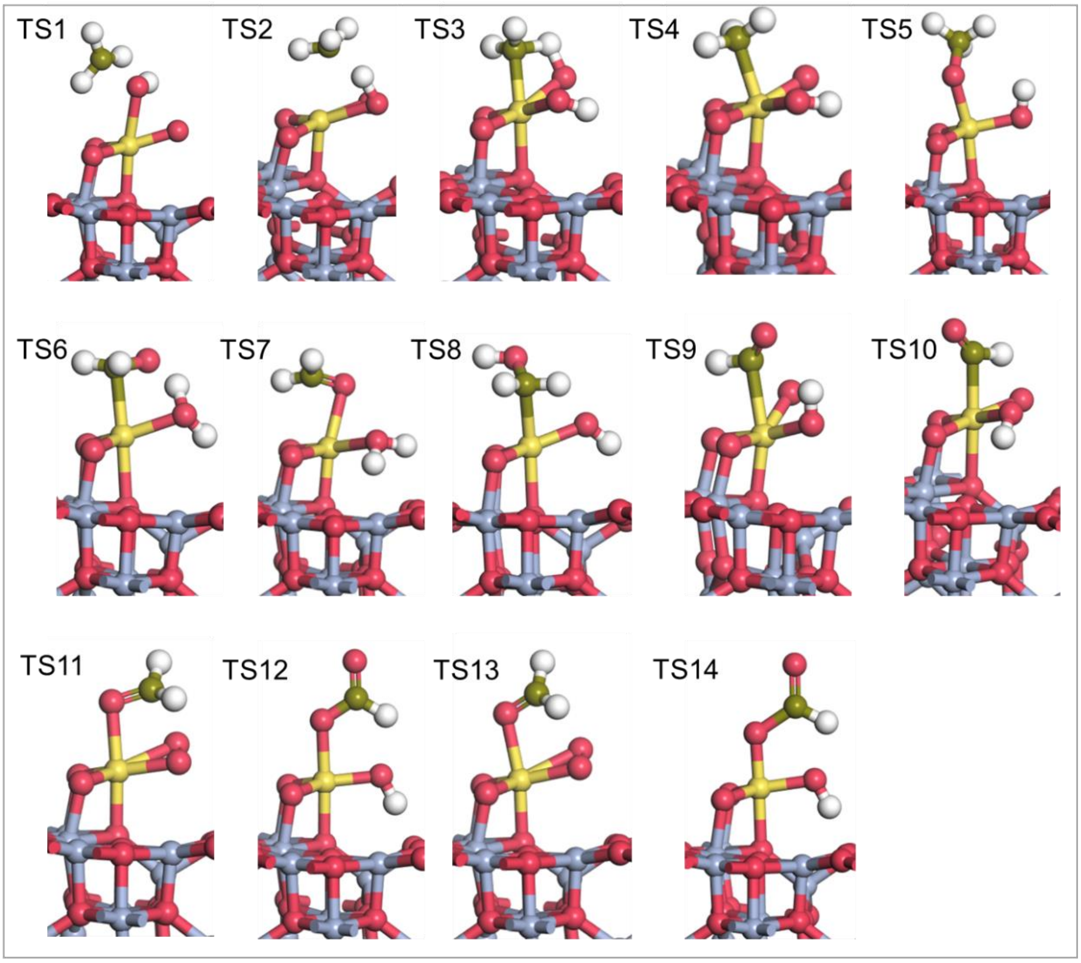

Supplementary Figure 32. Structural illustration of the transition state species. The gray, red, yellow, white, and brown spheres represent Al, O, Pd, H and C atoms, respectively.

Supplementary Note 24: Within the framework of the reaction mechanism network illustrated in Figure 5 of the main text, we undertook an investigation aimed at identifying transition state species associated with the conversions occurring between individual intermediates. Notably, it was discovered that specific elementary processes exhibit distinct transition states, denoted as TS1-14 in Figure 5, alongside their respective relative Gibbs free energies. The structures of these transition state species are depicted in Supplementary Figure 32.

Supplementary References

1. Yang, W.; Polo-Garzon, F.; Zhou, H.; Huang, Z.; Chi, M.; Meyer III, H.; Yu, X.; Li, Y.; Wu, Z., Boosting the activity of Pd single atoms by tuning their local environment on ceria for methane combustion. *Angewandte Chemie International Edition* **2023,** *62* (5), e202217323.
2. Yang, J.; Peng, M.; Ren, G.; Qi, H.; Zhou, X.; Xu, J.; Deng, F.; Chen, Z.; Zhang, J.; Liu, K., A hydrothermally stable irreducible oxide-modified Pd/MgAl2O4 catalyst for methane combustion. *Angewandte Chemie* **2020,** *132* (42), 18680-18684.
3. Petrov, A. W.; Ferri, D.; Krumeich, F.; Nachtegaal, M.; van Bokhoven, J. A.; Kröcher, O., Stable complete methane oxidation over palladium based zeolite catalysts. *Nature communications* **2018,** *9* (1), 2545.
4. Jiang, D.; Wan, G.; Halldin Stenlid, J.; García-Vargas, C. E.; Zhang, J.; Sun, C.; Li, J.; Abild-Pedersen, F.; Tassone, C. J.; Wang, Y., Dynamic and reversible transformations of subnanometre-sized palladium on ceria for efficient methane removal. *Nature Catalysis* **2023,** *6* (7), 618-627.
5. Xiong, H.; Kunwar, D.; Jiang, D.; García-Vargas, C. E.; Li, H.; Du, C.; Canning, G.; Pereira-Hernandez, X. I.; Wan, Q.; Lin, S., Engineering catalyst supports to stabilize PdOx two-dimensional rafts for water-tolerant methane oxidation. *Nature Catalysis* **2021,** *4* (10), 830-839.
6. Li, Z.; Xiao, Y.; Chowdhury, P. R.; Wu, Z.; Ma, T.; Chen, J. Z.; Wan, G.; Kim, T.-H.; Jing, D.; He, P., Direct methane activation by atomically thin platinum nanolayers on two-dimensional metal carbides. *Nature Catalysis* **2021,** *4* (10), 882-891.
7. Busca, G.; Lorenzelli, V., Infrared spectroscopic identification of species arising from reactive adsorption of carbon oxides on metal oxide surfaces. *Materials Chemistry* **1982,** *7* (1), 89-126.
8. Davydov, A. A.; Rochester, C. H., *Infrared spectroscopy of adsorbed species on the surface of transition metal oxides*. Wiley: 1990.
9. Almusaiteer, K.; Chuang, S. S., Dynamic behavior of adsorbed NO and CO under transient conditions on Pd/Al2O3. *Journal of Catalysis* **1999,** *184* (1), 189-201.
10. Kwak, J. H.; Hu, J.; Mei, D.; Yi, C.-W.; Kim, D. H.; Peden, C. H.; Allard, L. F.; Szanyi, J., Coordinatively unsaturated Al3+ centers as binding sites for active catalyst phases of platinum on γ-Al2O3. *Science* **2009,** *325* (5948), 1670-1673.
11. Zhang, Z.; Zhu, Y.; Asakura, H.; Zhang, B.; Zhang, J.; Zhou, M.; Han, Y.; Tanaka, T.; Wang, A.; Zhang, T., Thermally stable single atom Pt/m-Al2O3 for selective hydrogenation and CO oxidation. *Nature communications* **2017,** *8* (1), 16100.
12. Gao, F.; Gao, S.; Meng, S., Screening single-atom catalysts for methane activation: α− A l 2 O 3 (0001)-supported Ni. *Physical Review Materials* **2017,** *1* (3), 035801.
13. Kwak, J. H.; Hu, J.; Lukaski, A.; Kim, D. H.; Szanyi, J.; Peden, C. H., Role of pentacoordinated

Al3+ ions in the high temperature phase transformation of γ-Al2O3. *The Journal of Physical Chemistry C* **2008,** *112* (25), 9486-9492.
14. Khivantsev, K.; Jaegers, N. R.; Kwak, J. H.; Szanyi, J.; Kovarik, L., Precise Identification and Characterization of Catalytically Active Sites on the Surface of γ-Alumina. *Angewandte Chemie* **2021,** *133* (32), 17663-17671.
15. Zhu, X.; Wang, T.; Xu, Z.; Yue, Y.; Lin, M.; Zhu, H., Pt-Sn clusters anchored at Al3+ penta sites as a sinter-resistant and regenerable catalyst for propane dehydrogenation. *Journal of Energy Chemistry* **2022,** *65*, 293-301.
16. Çılgı, G.; Cetişli, H., Thermal decomposition kinetics of aluminum sulfate hydrate. *Journal of thermal analysis and calorimetry* **2009,** *98* (3), 855-861.
17. Chen, F.; Davis, J.; Fripiat, J., Aluminum coordination and Lewis acidity in transition aluminas. *Journal of Catalysis* **1992,** *133* (2), 263-278.
18. Rocha, J., Single-and triple-quantum 27Al MAS NMR study of the thermal transformation of kaolinite. *The Journal of Physical Chemistry B* **1999,** *103* (44), 9801-9804.
19. Sing, K. S., Reporting physisorption data for gas/solid systems with special reference to the determination of surface area and porosity (Recommendations 1984). *Pure and applied chemistry* **1985,** *57* (4), 603-619.
20. Szanyi, J.; Kuhn, W. K.; Goodman, D. W., CO adsorption on Pd (111) and Pd (100): Low and high pressure correlations. *Journal of Vacuum Science & Technology A: Vacuum, Surfaces, and Films* **1993,** *11* (4), 1969-1974.
21. Zhang, F.; Pan, L.; Li, T.; Diulus, J. T.; Asthagiri, A.; Weaver, J. F., CO oxidation on PdO (101) during temperature-programmed reaction spectroscopy: role of oxygen vacancies. *The Journal of Physical Chemistry C* **2014,** *118* (49), 28647-28661.
22. Dicks, O. A.; Shluger, A. L., Theoretical modeling of charge trapping in crystalline and amorphous Al2O3. *Journal of Physics: Condensed Matter* **2017,** *29* (31), 314005.
23. Lid, S.; Köppen, S.; Ciacchi, L. C., Creation of models and parametrization of a classical force field for amorphous Al2O3/water interfaces based on Density Functional Theory. *Computational Materials Science* **2017,** *140*, 307-314.
24. Digne, M.; Sautet, P.; Raybaud, P.; Euzen, P.; Toulhoat, H., Use of DFT to achieve a rational understanding of acid–basic properties of γ-alumina surfaces. *Journal of Catalysis* **2004,** *226* (1), 54-68.
25. Digne, M.; Sautet, P.; Raybaud, P.; Euzen, P.; Toulhoat, H., Hydroxyl groups on γ-alumina surfaces: a DFT study. *Journal of Catalysis* **2002,** *211* (1), 1-5.
26. Wischert, R.; Laurent, P.; Copéret, C.; Delbecq, F.; Sautet, P., γ-Alumina: the essential and unexpected role of water for the structure, stability, and reactivity of "defect" sites. *Journal of the*

American Chemical Society **2012,** *134* (35), 14430-14449.

27. Mei, D.; Kwak, J. H.; Hu, J.; Cho, S. J.; Szanyi, J.; Allard, L. F.; Peden, C. H., Unique role of anchoring penta-coordinated Al3+ sites in the sintering of γ-Al2O3-supported Pt catalysts. *The Journal of Physical Chemistry Letters* **2010,** *1* (18), 2688-2691.

28. Trinchero, A.; Hellman, A.; Grönbeck, H., Methane oxidation over Pd and Pt studied by DFT and kinetic modeling. *Surface science* **2013,** *616*, 206-213.

29. Mayernick, A. D.; Janik, M. J., Methane oxidation on Pd–Ceria: A DFT study of the mechanism over PdxCe1− xO2, Pd, and PdO. *Journal of Catalysis* **2011,** *278* (1), 16-25.

30. Stotz, H.; Maier, L.; Boubnov, A.; Gremminger, A.; Grunwaldt, J.-D.; Deutschmann, O., Surface reaction kinetics of methane oxidation over PdO. *Journal of Catalysis* **2019,** *370*, 152-175.

31. Jørgensen, M.; Gronbeck, H., First-principles microkinetic modeling of methane oxidation over Pd (100) and Pd (111). *ACS Catalysis* **2016,** *6* (10), 6730-6738.

32. Antony, A.; Asthagiri, A.; Weaver, J. F., Pathways and kinetics of methane and ethane C–H bond cleavage on PdO (101). *The Journal of chemical physics* **2013,** *139* (10), 104702.
